# Decoding the Neural Circuit Underlying Context‐Cue Association in Retrieval of Morphine Reward Memory

**DOI:** 10.1002/advs.77735

**Published:** 2026-09-16

**Authors:** Zhifeng Shi, Guangying Li, Qian Yang, Rusun Liu, Linghan Gu, Mingmei Wang, John L. Waddington, Fuhai Ji, Xuechu Zhen

**Affiliations:** ^1^ Department of Anesthesiology First Affiliated Hospital of Soochow University Suzhou Jiangsu China; ^2^ Jiangsu Key Laboratory of Drug Discovery and Translational Research For Brain Diseases College of Pharmaceutical Sciences Soochow University Suzhou Jiangsu China; ^3^ Institute of Anesthesiology Soochow University Suzhou Jiangsu China; ^4^ College of Biology & Food Sciences Suzhou University of Technology Suzhou Jiangsu China; ^5^ School of Pharmacy and Biomolecular Sciences RCSI University of Medicine and Health Sciences Dublin Ireland

**Keywords:** dmPFC, glutamatergic projection, microcircuit, reward memory retrieval, vCA1

## Abstract

Context‐induced relapse is a major challenge in treating drug use disorders, yet the neural circuits underlying drug memory retrieval remain unclear. A glutamatergic projection from the ventral CA1 (vCA1) to the dorsomedial prefrontal cortex (dmPFC) is identified as a critical pathway for morphine reward memory retrieval. In vivo monitoring and ex vivo electrophysiology show that vCA1 inputs engage distinct dmPFC neuronal populations and differentially regulate local parvalbumin (PV+) and somatostatin (SST+) interneurons, thereby reshaping cortical output during memory retrieval. Pathway‐specific inhibition abolishes cue‐induced memory retrieval, which is restored by local NMDA administration, whereas blockade of NMDA receptors in the dmPFC impairs retrieval during pathway activation, supporting a critical role for NMDA receptor signaling in memory retrieval driven by vCA1 input. Beyond reward memory, chronic inhibition of the vCA1–dmPFC pathway attenuates somatic withdrawal and the development of analgesic tolerance without affecting the acute analgesic effect of morphine. Together, these findings reveal a cell‐type‐specific hippocampal–prefrontal mechanism regulating opioid‐associated maladaptive behaviors and provide a potential circuit‐level strategy to reduce addiction‐related consequences while preserving morphine analgesia.

## Graphical abstract

Contextual cues recruit the glutamatergic vCA1–dmPFC pathway to retrieve morphine reward memory by reshaping cell‐type‐specific prefrontal microcircuit activity. Distinct regulation of PV+ and SST+ interneurons defines this hippocampal–prefrontal mechanism. Notably, pathway inhibition suppresses reward memory retrieval, somatic withdrawal, and analgesic tolerance while preserving the acute analgesic efficacy of morphine.

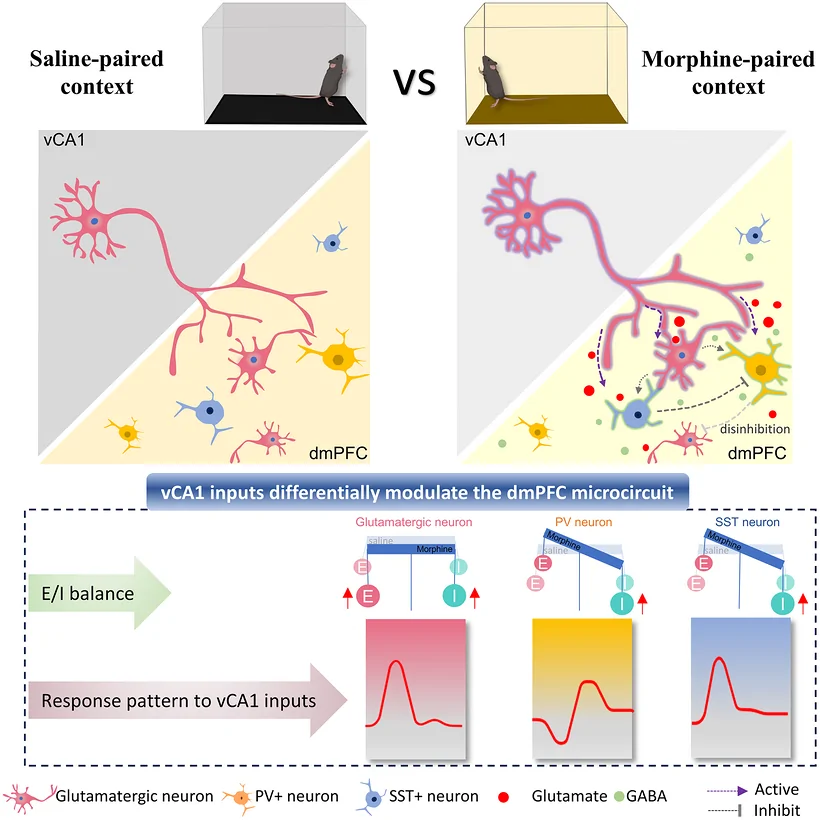

## 1 Introduction

Opioids remain the gold standard for clinical pain management, yet their therapeutic utility is severely limited by a strong liability for addiction and physical dependence [1, 2, 3, 4]. The transition to substance use disorder is fundamentally rooted in the hijacking of neural systems that govern learning and memory [5]. Retrieval of morphine‐associated reward memories is considered a critical driving force for dependence and relapse in drug abuse patients, including opioid users [6, 7, 8, 9]. Therefore, disrupting the associative memories that link environmental cues to drug experiences has emerged as a promising therapeutic strategy for drug use disorders [10, 11, 12, 13, 14, 15, 16]. The hippocampus, particularly its ventral CA1 subregion (vCA1), is considered a pivotal structure for mediating the retrieval of morphine reward memory due to its ability to integrate contextual information [17, 18, 19, 20, 21]. Glutamatergic vCA1 projection neurons densely innervate downstream limbic and cortical structures, including the medial prefrontal cortex (mPFC), nucleus accumbens (NAc), and basolateral amygdala (BLA) [18, 19, 22, 23, 24, 25]. Although acute drug reward is primarily mediated by the mesolimbic dopamine circuits, the long‐term, context‐cue‐driven drug memory and relapse depend more on broader neural network activities, including cortical and limbic structures [26, 27]. Given that glutamatergic neurons constitute the major excitatory population in these networks and exhibit remarkable structural and functional responses to environmental stimuli [28, 29], they are thought to be primary targets for memory retrieval and major mediators of addiction‐related pathophysiology. Indeed, glutamatergic neurotransmission is essential for the formation and maintenance of these drug memories and drives cue‐induced relapse [30]. However, how hippocampal glutamatergic afferents dynamically orchestrate cortical microcircuits during memory retrieval remains poorly understood.

Stable cortical computation depends on the precise and dynamic coordination of excitation and inhibition (E/I balance), mediated by glutamatergic pyramidal neurons (PNs) and diverse GABAergic interneurons (INs) [22, 31, 32]. Psychostimulants and opioids perturb cortico‐subcortical networks and drive structural and functional synaptic plasticity and are thereby consequential for cue‐driven behaviors [33, 34]. However, the precise contributions of PNs and GABAergic INs within mPFC to drug reward memory are still debated. While some studies report that chemogenetic silencing of mPFC PNs attenuates drug‐seeking, others demonstrate that prefrontal hypoactivity promotes addiction and that restoring PN firing suppresses relapse [35, 36]. We propose that this apparent paradox arises from a failure to resolve the underlying microcircuit dynamics.

Within the mPFC, cortical activity is tightly gated by distinct interneuron subtypes, most prominently parvalbumin‐expressing (PV+) and somatostatin‐expressing (SST+) interneurons. These populations exert complementary spatial and temporal inhibitory control over pyramidal neurons and are essential for maintaining cortical E/I balance [22, 30, 35, 36, 37]. PV+ interneurons provide rapid perisomatic inhibition that constrains pyramidal neuron output, whereas SST+ interneurons regulate dendritic integration and frequently participate in disinhibitory microcircuits [37, 38, 39]. Despite the importance of these inhibitory motifs in cortical computation, whether long‐range contextual signals from the hippocampus selectively recruit specific interneuron populations to gate drug memory retrieval remains unknown.

In this study, we define the cellular and microcircuit mechanisms by which the vCA1–dmPFC pathway governs opioid reward memory retrieval. Using in vivo neurotransmitter sensing, ex vivo electrophysiology, and intersectional viral strategies, we show that cue‐induced memory retrieval is not mediated by a simple excitatory relay but relies on glutamate‐dependent changes in dmPFC network activity. vCA1 inputs modulate local synaptic dynamics through cell‐type‐specific regulation of dmPFC glutamatergic neurons and inhibitory interneurons, including PV+ and SST+ populations, thereby shaping the cortical network status associated with memory expression. Silencing this pathway abolishes morphine reward memory retrieval and markedly attenuates somatic withdrawal and analgesic tolerance while preserving acute analgesic efficacy of morphine. Together, these findings identify a hippocampal–prefrontal circuit that contributes to opioid‐associated memory processes and chronic opioid adaptations, suggesting a potential strategy to reduce addictive risk while maintaining effective analgesia.

## 2 Results

### 2.1 Excitatory vCA1 Outputs to dmPFC Convey Distinct Contextual Information in Morphine Reward Memory

**FIGURE 1:**
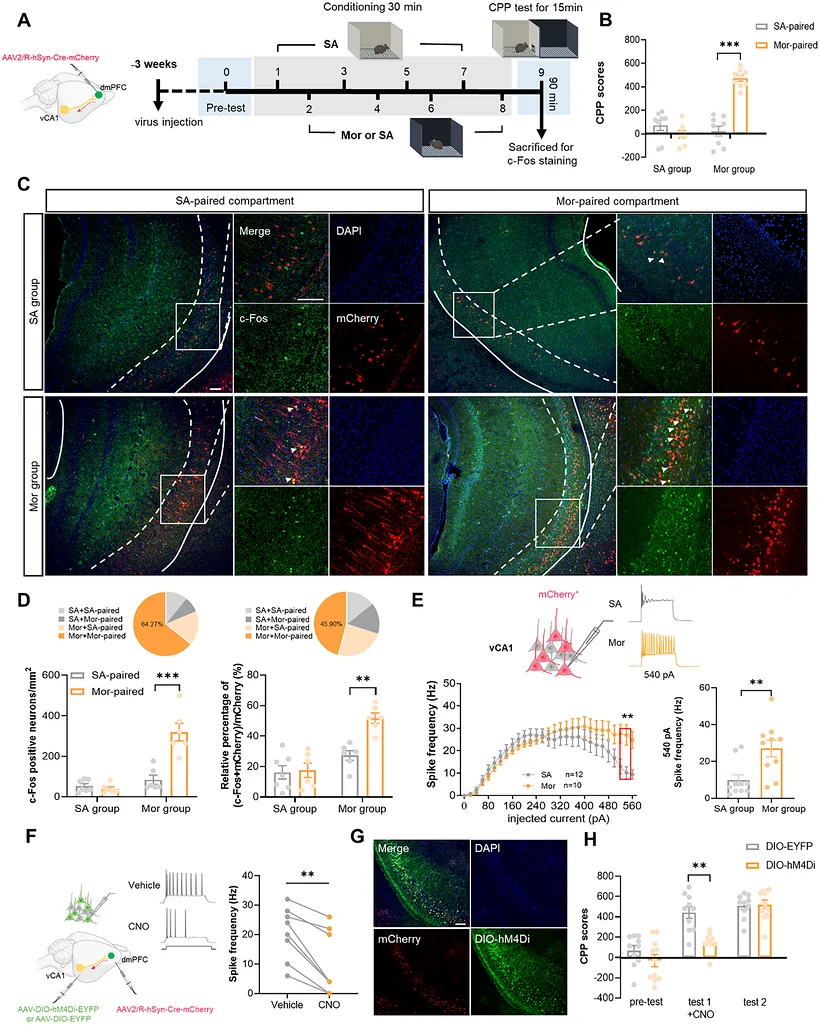
Conditioned context increases c‐Fos expression in vCA1 neurons projecting to dmPFC in mice with morphine reward memory. (A) Schematic of retrograde tracing from dmPFC to vCA1 (left) and experimental timeline for the morphine CPP procedure (right). Mice were divided into saline‐conditioned (SA) and morphine‐conditioned (Mor) groups. In the SA group, both compartments were paired with saline. In the Mor group, one compartment was paired with morphine and the other with saline. (B) Morphine‐conditioned mice exhibited a robust preference for the morphine (Mor)‐paired compartment compared with saline (SA)‐conditioned controls (SA group: saline‐paired vs. saline‐paired compartments; Mor group: saline‐paired vs. morphine‐paired compartments; SA‐conditioned group: n = 8 each; Mor‐conditioned group: n = 9 each; *F*_(1, 30)_ = 53.62, *p* < 0.001, two‐way ANOVA). (C) Representative images showing c‐Fos expression (green) and mCherry+ labeled vCA1 neurons projecting to dmPFC. Scale bars: 100 µm. (D) Quantification of c‐Fos+ cell density in vCA1 (left) and percentage of c‐Fos+ /mCherry+ double‐labeled neurons relative to total mCherry+ neurons across groups (right) (SA‐conditioned group: saline‐paired vs. saline‐paired compartments, n = 7; Mor‐conditioned group: saline‐paired vs. morphine‐paired compartments, n = 6 each, left: *F*_(1, 21)_ = 25.24, *p* < 0.001; right: *F*_(1, 21)_ = 8.23, *p* = 0.009, two‐way ANOVA). (E) Schematic of ex vivo whole‐cell patch‐clamp recording from mCherry+ vCA1–dmPFC projection neurons (left); representative traces (middle) and quantification of spike numbers (right) in Mor‐ and SA‐conditioned mice (SA group: 12 cells from 3 mice; Mor group: 10 cells from 3 mice; spike number was analyzed across different current injection steps, *F*_(28, 532)_ = 3.75, *p* = 0.005, two‐way ANOVA). (F) Schematic of chemogenetic inhibition of dmPFC‐projecting Glu^vCA1^ neurons (left); CNO incubation (5 µM) significantly reduced action potential firing in DIO‐hM4Di‐expressing neurons in response to 80 pA depolarizing current injection (n = 8 cells from 2 mice, tested before and after CNO application, *t*_(7)_ = 5.27, *p* = 0.001, paired Student's *t*‐test). (G) Representative images showing co‐localization of mCherry+ with DIO‐hM4Di‐expressing neurons in vCA1. Scale bars: 100 µm. (H) Chemogenetic inhibition of dmPFC‐projecting Glu^vCA1^ neurons significantly reduced time spent in the Mor‐paired compartment (DIO‐EYFP group: n = 10; DIO‐hM4Di group: n = 11; *F*_(2, 38)_ = 8.61, *p* < 0.001, two‐way ANOVA). Data are presented as mean ± SEM; ^**^ *p* < 0.01, ^***^ *p* < 0.001.

To investigate how conditioned cues activate excitatory projections from ventral CA1 (vCA1) to dmPFC in mediating retrieval of morphine‐associated reward memory, we first examined c‐Fos expression in vCA1 neurons in mice subjected to a morphine‐conditioned place preference (CPP) paradigm. A retrograde viral tracer (retroAAV‐hSyn‐mCherry) was injected into dmPFC to label vCA1 neurons projecting to dmPFC three weeks before application of the morphine‐induced CPP paradigm (Figure 1A,B). Re‐exposure to the morphine‐paired context elicited a significant increase in c‐Fos in vCA1. Notably, a greater number of c‐Fos‐positive neurons (c‐Fos+) were co‐localized with mCherry‐positive neurons (mCherry+), indicating that dmPFC‐projecting vCA1 neurons are preferentially recruited during morphine memory retrieval (Figure 1C,D). Strong mCherry+ signals were further confirmed in vCA1 glutamatergic neurons (Glu^vCA1^, Figure S1A). Furthermore, ex vivo whole‐cell patch‐clamp recordings revealed that chronic morphine exposure significantly enhanced the intrinsic excitability of these dmPFC‐projecting vCA1 neurons in response to higher‐intensity current injections (Figure 1E). Collective anatomical and electrophysiological data demonstrated that conditioned contextual cues selectively activate the excitatory vCA1–dmPFC projection following the establishment of morphine reward memory.

To assess the functional role of this pathway, AAV‐CaMKIIα‐ChR2‐EYFP or AAV‐CaMKIIα‐NpHR‐mCherry were injected into vCA1, with optical fibers implanted in the dmPFC. Using a real‐time place preference (RTPP) paradigm, we tested whether optogenetic manipulation of the Glu^vCA1^–dmPFC circuit drives innate reward or aversion (Figure S1B,D). As shown in Figure S1C, neither activation nor inhibition of vCA1 terminals in the dmPFC altered avoidance or preference behaviors in mice exposed to the photostimulation‐paired compartment when compared with those of controls. In order to identify the specific role of Glu^vCA1^–dmPFC projections in context‐induced retrieval of morphine reward memory, we expressed NpHR or hM4Di in dmPFC‐projecting vCA1 neurons. Both optogenetic and chemogenetic approaches to silence the Glu^vCA1^–dmPFC pathway significantly attenuated morphine CPP scores during the manipulation phase (Test 1). Importantly, this impairment was transient and reversible, as memory expression remained intact during a subsequent manipulation‐free session (Test 2) (Figure S2). Similarly, by injecting retroAAV‐Cre into the dmPFC and AAV‐DIO‐hM4Di‐EYFP into the vCA1, we confirmed that chemogenetic inhibition of strictly projection‐defined vCA1 neurons also reduced CPP scores in Test 1 but not in Test 2 (Figure 1F–H).

Notably, inhibition of vCA1 outputs to dmPFC neurons did not alter basal activity or general cognitive performance, as this manipulation failed to alter general learning and memory in the novel object recognition (NOR) test nor locomotor activity in the open field test (OFT) (Figure S3A–C). These findings demonstrate that excitatory projections from vCA1 to dmPFC are critical for retrieval of morphine reward memory, independent of generalized motor or innate valence effects.

### 2.2 Concurrent Engagement of vCA1 and dmPFC Glutamatergic Neurons is Required for Morphine Reward Memory Retrieval

**FIGURE 2:**
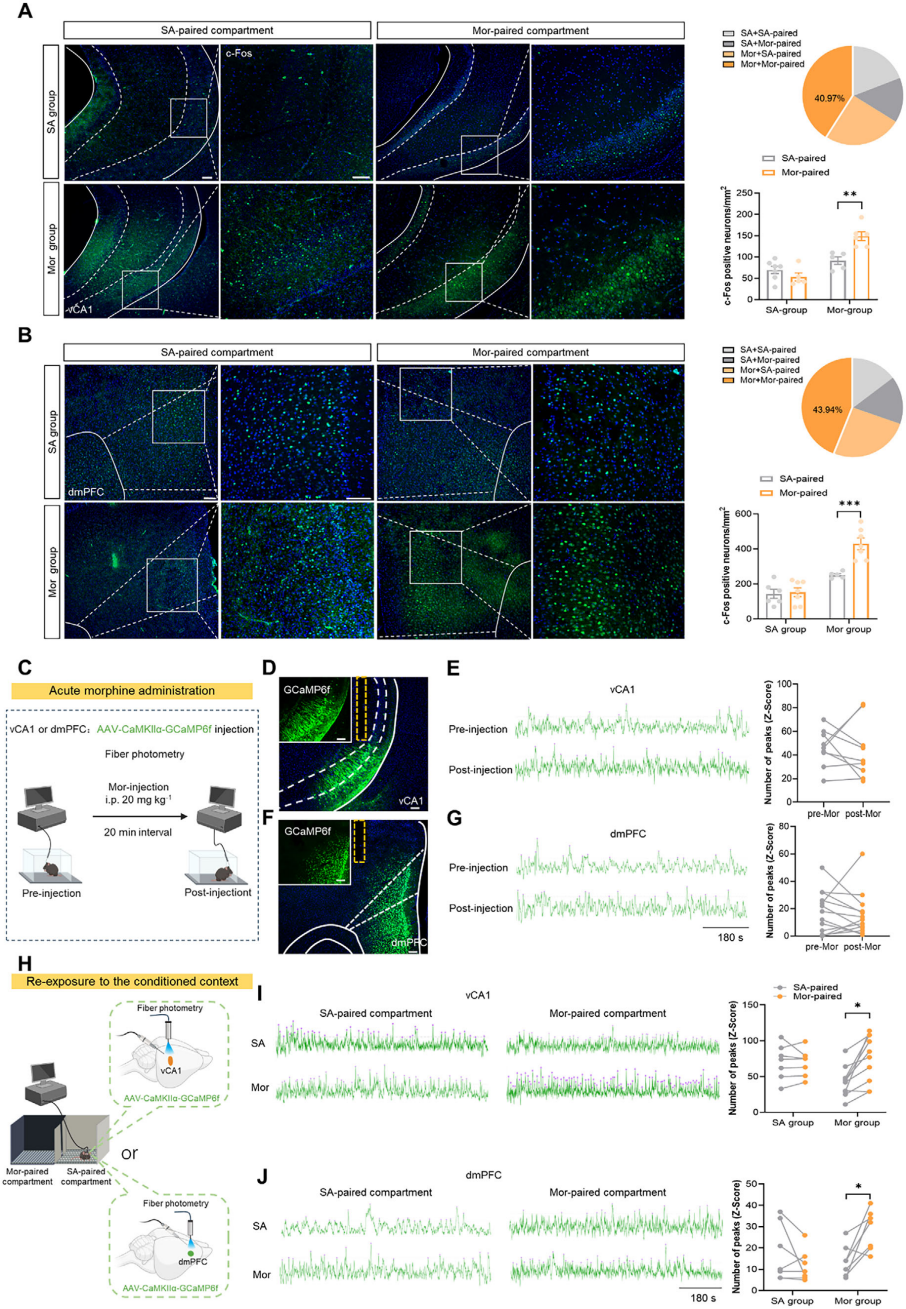
Conditioned contextual cues activate glutamatergic neuronal activity in both vCA1 and dmPFC. (A,B) Representative image (left) and quantification (right) of c‐Fos+ cells in vCA1 and dmPFC of SA‐ and Mor‐conditioned mice (vCA1: SA‐conditioned group: saline‐paired vs. saline‐paired compartments, n = 7; Mor‐conditioned group: saline‐paired vs. morphine‐paired compartments, n = 5 each; Mor‐conditioned group: saline‐paired and morphine‐paired compartment, n = 6; *F*_(1, 19)_ = 14.76, *p* = 0.001; dmPFC: SA‐conditioned group: saline‐paired vs. saline‐paired compartments, n = 6; Mor‐conditioned group: saline‐paired and morphine‐paired compartments, n = 7, each; Mor + SA‐paired group, n = 5; *F*_(1, 21)_ = 9.82, *p* = 0.005, two‐way ANOVA). Scale bars: 100 µm. (C) Schematic for fiber photometry recordings following acute morphine administration. (D,F) Representative fluorescence images showing GCaMP6f expression in vCA1 (top) and dmPFC (bottom). Scale bars: 100 µm. (E,G) Representative traces (left) showing effective Ca^2+^ signal peaks of vCA1 (E) or dmPFC (G) glutamatergic neurons and quantification of calcium transient peaks (right) in saline‐ vs. acute morphine‐treated mice (vCA1: n = 9, *t*_(8)_ = 0.31, *p* = 0.77; dmPFC: n = 13, *t*_(12)_ = 0.48, *p* = 0.64, paired Student's *t*‐test). (H) Schematic diagram of fiber photometry recordings during re‐exposure to the conditioned context following CPP training. (I,J) Representative Ca^2^^+^ transients (left) and quantification (right) in vCA1 (I) and dmPFC (J) across different groups (vCA1: SA‐conditioned group: saline‐paired compartments, n = 8; Mor‐conditioned group: saline‐paired and morphine‐paired compartments, n = 10 each; *F*_(1, 32)_ = 4.11, *p* = 0.05; dmPFC: SA‐conditioned group: saline‐paired compartments, n = 7; Mor‐conditioned group: saline‐paired and morphine‐paired compartments, n = 7 each; *F*_(1, 24)_ = 7.75, *p* = 0.01, two‐way ANOVA). Data are presented as mean ± SEM; ^*^ *p* < 0.05, ^**^ *p* < 0.01, ^***^ *p* < 0.001.

We next examined whether morphine reward memory retrieval involves concurrent activation of Glu^vCA1^ and dmPFC glutamatergic neurons (Glu^dmPFC^). As shown in Figure 2A,B, re‐exposure to the conditioned contextual cues elicited a significant upregulation of c‐Fos expression within both regions. To monitor the real‐time in vivo dynamics of these excitatory networks, we expressed CaMKIIα‐driven GCaMP6f in either vCA1 or dmPFC. Following acute morphine administration, no significant changes in GCaMP6f fluorescence were observed in either brain region, suggesting that a single morphine exposure did not alter regional glutamatergic neuronal activity (Figure 2C–G). In contrast, re‐exposure to the conditioned context in morphine CPP mice induced a robust increase in GCaMP6f fluorescence in both vCA1 and dmPFC, indicating that context‐driven memory retrieval, but not acute pharmacological exposure, recruits glutamatergic populations in the two regions. (Figure 2H–J).

**FIGURE 3:**
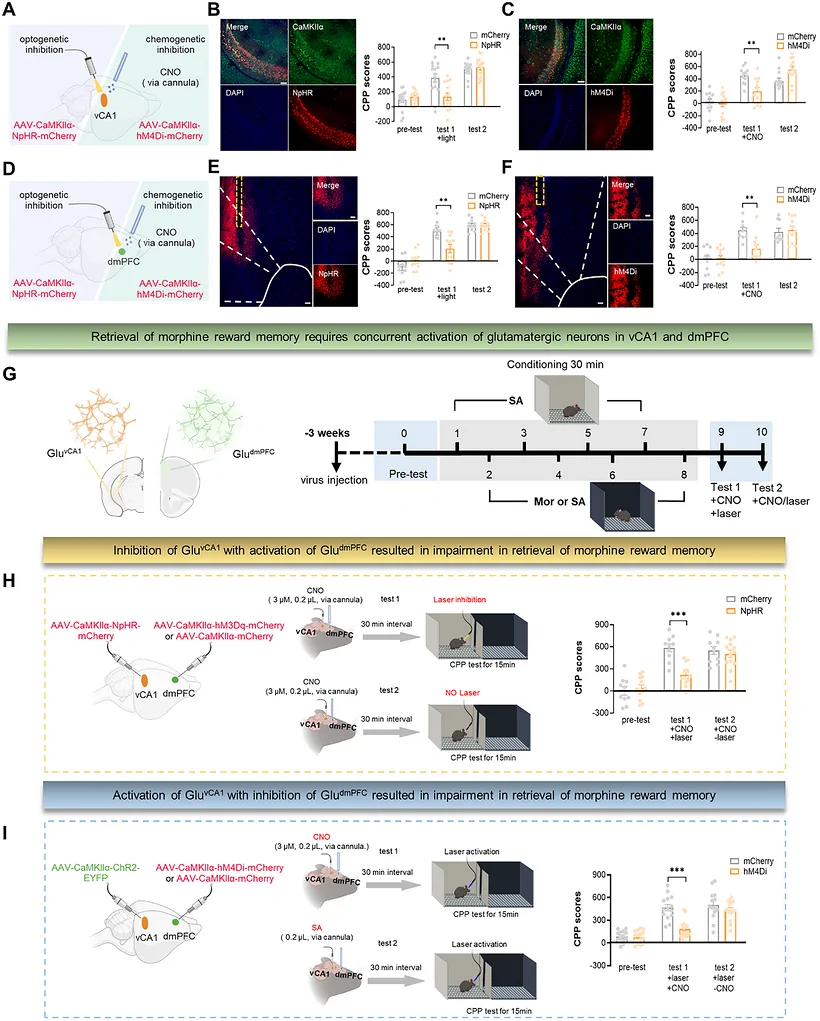
Retrieval of morphine reward memory depends on coordinated activity of glutamatergic neurons in vCA1 and dmPFC. (A,D) Schematic illustration of optogenetic and chemogenetic targeting of glutamatergic neurons in vCA1 or dmPFC. (B,E) Representative images (left) showing NpHR expression in vCA1 (B) and dmPFC (E). Optogenetic silencing (right) in either region significantly reduced time mice spent in the morphine‐paired compartment (vCA1: mCherry group, n = 15; NpHR group, n = 14; *F*_(2, 54)_ = 8.52, *p* < 0.001; dmPFC: mCherry group, n = 15; NpHR group, n = 15; *F*_(2, 60)_ = 8.68, *p* < 0.001, two‐way ANOVA). Scale bars: 100 µm. (C, F) Representative images (left) showing hM4Di expression in vCA1 (C) and dmPFC (F) and quantification (right) of CPP performance. Chemogenetic silencing also significantly impaired morphine memory retrieval (vCA1: n = 12 per group; *F*_(2, 66)_ = 9.87, *p* < 0.001; dmPFC: mCherry group, n = 9; hM4Di group, n = 11; *F*_(2, 54)_ = 4.78, *p* = 0.012, two‐way ANOVA). Scale bars: 100 µm. (G) Experimental timeline for CPP procedure. (H, I) Schematic illustrations of cross‐region manipulations (left) and quantification of CPP performance (right). Optogenetic inhibition of Glu^vCA1^ combined with chemogenetic activation of Glu^dmPFC^ (H) or optogenetic activation of Glu^vCA1^ combined with chemogenetic inhibition of Glu^dmPFC^ (I) both significantly impaired memory retrieval despite excitatory activation in one region, indicating the necessity of coordinated vCA1 and dmPFC activity (H: mCherry group, n = 10; NpHR group, n = 13; *F*_(2, 63)_ = 9.36, *p* < 0.001; I: mCherry group, n = 14; hM4Di group, n = 15; *F*_(2, 81)_ = 8.81, *p* < 0.001, two‐way ANOVA). Data are presented as mean ± SEM; ^**^ *p* < 0.01, ^***^ *p* < 0.001.

To further dissect the role of glutamatergic activation, we then silenced glutamatergic neurons by expressing NpHR or hM4Di under CaMKIIα promoter control (Figure 3A,D). Optogenetic or chemogenetic inhibition of Glu^vCA1^ or Glu^dmPFC^ neurons significantly reduced the time mice spent in the morphine‐paired compartment, confirming that local excitatory activity in either region is indeed required for reward memory retrieval (Figure 3B,C,E,F). To further determine whether these two regions can compensate for one another during morphine reward memory retrieval, a dual regional manipulation approach was employed (Figure 3G–I). Strikingly, chemogenetic activation of Glu^dmPFC^ (hM3Dq) failed to rescue the memory retrieval deficit caused by simultaneous optogenetic silencing (NpHR) of Glu^vCA1^ (Figure 3H). Furthermore, optogenetic stimulation of Glu^vCA1^ (ChR2) also failed to restore morphine‐associated reward memory when Glu^dmPFC^ neurons were chemogenetically inhibited (hM4Di) (Figure 3I). These findings indicate that activation of either region alone is insufficient to compensate for the functional deficit of the other, suggesting a functional interdependence between vCA1 and dmPFC glutamatergic populations during reward memory retrieval.

Importantly, these targeted interventions did not broadly compromise basal behavioral output. Inhibition of Glu^vCA1^ did not affect the basal recognition memory or locomotor activity of mice (Figure S4A,B). Although chemogenetic inhibition of Glu^dmPFC^ induced a modest reduction in total locomotion, it did not cause broad motor or cognitive deficits that could fully account for the profound loss of CPP expression (Figure S4C,D).

### 2.3 PV+ and SST+ Interneurons Differentially Regulate Morphine Reward Memory Retrieval

**FIGURE 4:**
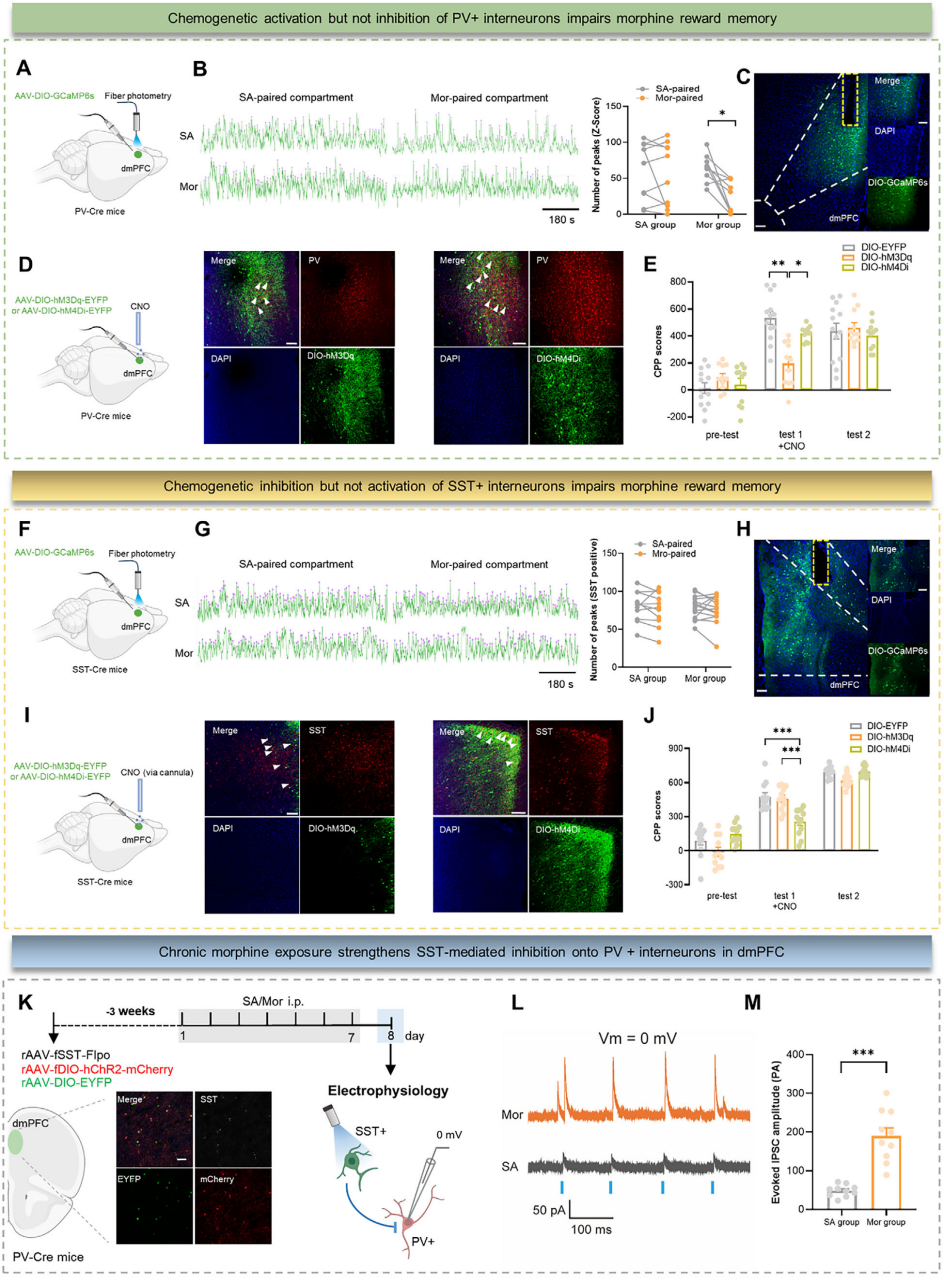
PV+ and SST+ interneurons in the dmPFC differentially contribute to retrieval of morphine reward memory. (A,F) Schematic illustration of fiber photometry recordings in PV+ or SST+ interneurons of the dmPFC. (B,G) Representative Ca^2+^ traces (left) and quantification of calcium transient peaks (right) in PV+ (B) and SST+ interneurons (G) during re‐exposure to the morphine‐paired context. PV+ interneurons showed significantly decreased activity, whereas SST+ interneurons remained unaffected (PV+: SA group, n = 10; Mor group, n = 9; *F*_(1, 34)_ = 4.22, *p* = 0.048; SST+: SA group, n = 11; Mor group, n = 14; *F*_(1, 26)_ = 0.25, *p* = 0.62, two‐way ANOVA). (C, H) Representative fluorescence images showing DIO‐GCaMP6s expression in dmPFC PV+ (C) and SST+ interneurons (H). Scale bars: 100 µm. (D, I) Schematic illustration of chemogenetic manipulation in PV+ (D) or SST+ interneurons (I) (left) and representative fluorescence images confirming DIO‐EYFP expression (right). Scale bars: 100 µm. (E) Chemogenetic activation, but not inhibition, of PV+ interneurons significantly reduced CPP scores (DIO‐EYFP group, n = 13; DIO‐hM3Dq, DIO‐hM4Di groups, n = 11 each; *F*_(4, 96)_ = 7.88, *p* < 0.001, two‐way ANOVA). (J) Chemogenetic inhibition, but not activation, of SST+ interneurons significantly reduced CPP scores (DIO‐EYFP, DIO‐hM4Di groups, n = 12 each; DIO‐hM3Dq group, n = 11; *F*_(4, 96)_ = 11.72, *p* < 0.001, two‐way ANOVA). (K) Schematic illustration of optogenetic activation of SST+ interneurons and whole‐cell patch‐clamp recordings from PV+ interneurons in the dmPFC, with representative fluorescence images confirming viral expression and cell‐type‐specific targeting. Scale bars: 100 µm. (L) Representative optogenetically evoked IPSC traces recorded from PV+ interneurons following SST+ interneuron stimulation in saline‐ and morphine‐treated mice. (M) Quantification of SST+ interneuron‐mediated inhibitory synaptic transmission onto PV+ interneurons. Chronic morphine exposure significantly increased the amplitude of evoked IPSCs compared with saline controls (SA group, n = 10 cells from 3 mice; Mor group, n = 10 cells from 3 mice; *t*_(18)_ = 6.55, *p* < 0.001, unpaired Student's *t*‐test). Data are presented as mean ± SEM; ^*^ *p* < 0.05, ^**^ *p* < 0.01, ^***^ *p* < 0.001.

PV+ and SST+ interneurons represent two major subtypes of inhibitory neurons in dmPFC (PV^dmPFC^, SST^dmPFC^). To elucidate their real‐time dynamics during the retrieval of morphine reward memory in vivo, we performed cell‐type‐specific fiber photometry recordings by bilaterally expressing AAV‐DIO‐GCaMP6s in the dmPFC of PV‐Cre and SST‐Cre mice (Figure 4A,F). Re‐exposure to the morphine‐paired compartment resulted in decreased calcium activity in PV^dmPFC^ neurons (Figure 4B,C). In contrast, SST^dmPFC^ exhibited no significant population‐level changes in calcium signals during the same retrieval phase (Figure 4G,H).

To determine the functional contribution of distinct interneuron populations to morphine reward memory retrieval, we next performed bidirectional chemogenetic manipulations by expressing excitatory (hM3Dq) or inhibitory (hM4Di) DREADDs in PV^dmPFC^ or SST^dmPFC^ neurons (Figure 4D,I). Chemogenetic activation of PV^dmPFC^ or inhibition of SST^dmPFC^ neurons significantly suppressed morphine CPP, whereas inhibition of PV^dmPFC^ or activation of SST^dmPFC^ neurons produced no significant effect (Figure 4E,J). We further examined the synaptic interaction between SST+ and PV+ interneurons using cell‐type‐specific ex vivo recordings. Optogenetic activation of SST^dmPFC^ neurons directly evoked inhibitory postsynaptic currents (IPSCs) in PV+ neurons, confirming a direct inhibitory SST–PV connection. Notably, SST‐mediated inhibition of PV+ neurons was significantly enhanced following repeated morphine exposure, as compared to that of saline controls (Figure 4K–M).

Similarly, we confirmed that these manipulations also did not affect general cognitive function as measured by the novel object recognition test (Figure S5A,C). However, activating PV^dmPFC^ or inhibiting SST^dmPFC^ neurons disrupted CPP and reduced basal locomotor activity in the open field test (Figure S5B,D).

### 2.4 vCA1 Inputs Differentially Modulate the dmPFC Microcircuit

**FIGURE 5:**
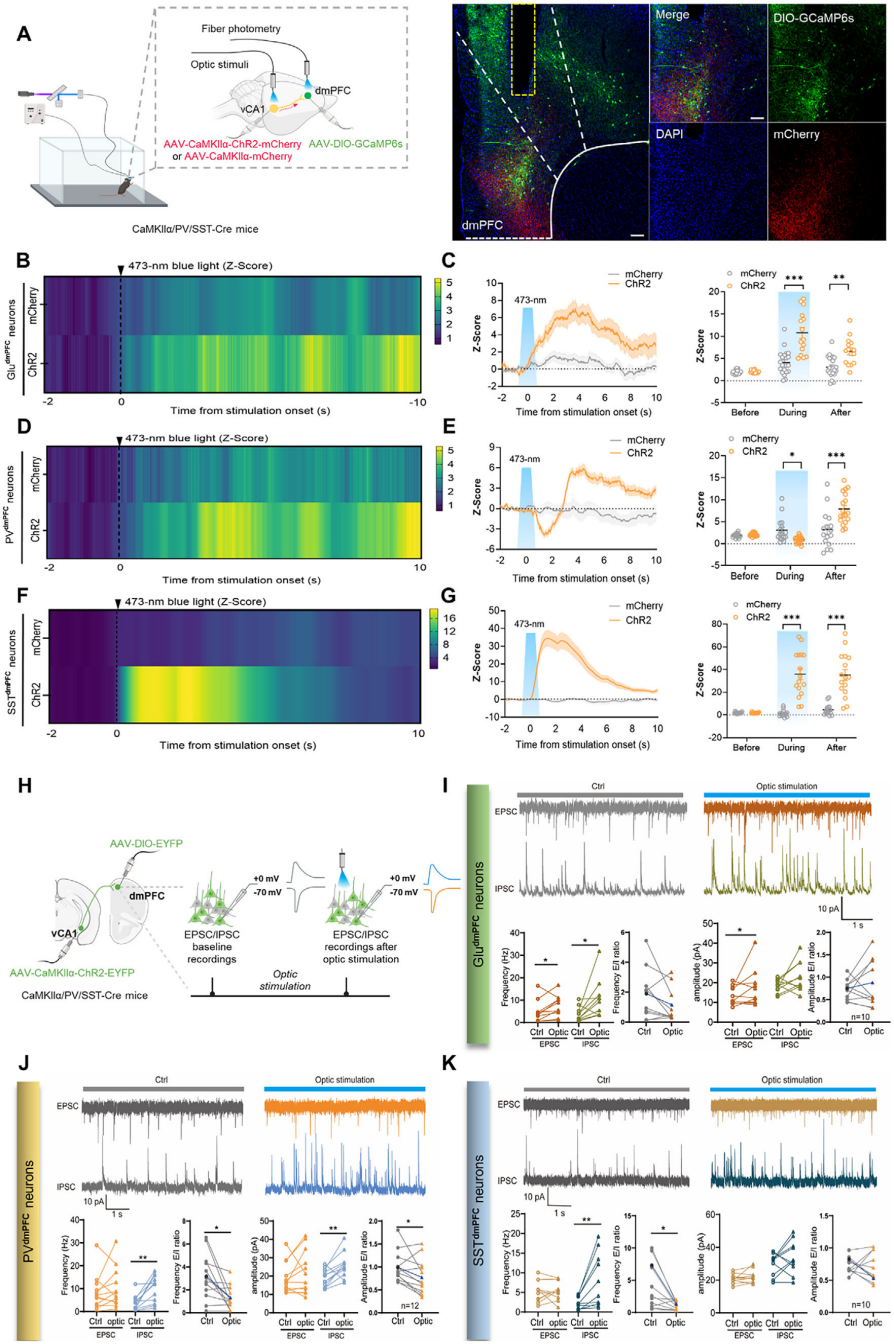
Circuit‐level dissection of glutamatergic vCA1 inputs onto distinct neuronal subtypes in dmPFC. (A) Schematic of experimental design for fiber photometry recording in genetically defined dmPFC neuronal subtypes during optogenetic activation of Glu^vCA1^ inputs (left). Representative fluorescence image showing DIO‐GCaMP6s expression in dmPFC neuronal subtypes and ChR2‐expressing Glu^vCA1^ axon terminals (right). Scale bars: 100 µm. (B,D,F) Heatmaps of Ca^2+^ signals aligned to photostimulation onset across trials in dmPFC glutamatergic neurons (B), PV‐INs (D), and SST‐INs (F). (C,E,G) Representative traces (left) and averaged z‐score (right) of Ca^2+^ signals in Glu^dmPFC^ (C), PV^dmPFC^ (E), and SST^dmPFC^ (G) neurons in mCherry‐and ChR2‐expressing mice. Bold line (orange for ChR2 and gray for mCherry) indicate mean values and shaded areas represent SEM (Glu^dmPFC^: mCherry group, n = 21; ChR2 group, n = 15; *F*_(2, 68)_ = 18.48, *p* < 0.001; PV^dmPFC^: mCherry group, n = 16; ChR2 group, n = 18; *F*_(2, 64)_ = 20.99, *p* < 0.001; SST^dmPFC^: mCherry group, ChR2 groups, n = 15 each; *F*_(2, 60)_ = 41.87, *p* < 0.001, two‐way ANOVA). (H) Schematic of simultaneous optogenetic activation of Glu^vCA1^ axon terminals and whole‐cell patch‐clamp recordings of EYFP‐labeled neurons in dmPFC to examine synaptic connectivity across neuronal subtypes. (I–K) Representative traces and summary graphs showing the effects of optogenetic activation of Glu^vCA1^ terminals on EPSC or IPSC frequency and amplitude, together with E/I ratio, in dmPFC glutamatergic neurons (I, 10 cells from 3 mice), PV+ interneurons (J, 12 cells from 3 mice) and SST+ interneurons (K, 10 cells from 3 mice) (Glu^dmPFC^‐EPSC frequency: *t*_(9)_ = 2.27, *p* = 0.049, Glu^dmPFC^‐IPSC frequency: *t*_(9)_ = 2.57, *p* = 0.03, Glu^dmPFC^‐EPSC amplitude: *t*_(9)_ = 2.58, *p* = 0.032, Glu^dmPFC^‐IPSC amplitude: *t*_(9)_ = 1.81, *p* = 0.104, Glu^dmPFC^‐ frequency E/I ratio: *t*_(9)_ = 1.70, *p* = 0.124, Glu^dmPFC^‐ amplitude E/I ratio: *t*_(9)_ = 0.86, *p* = 0.414; PV^dmPFC^‐EPSC frequency: *t*_(11)_ = 0.95, *p* = 0.37, PV^dmPFC^‐IPSC frequency: *t*_(11)_ = 4.26, *p* = 0.001, PV^dmPFC^‐EPSC amplitude: *t*_(11)_ = 1.18, *p* = 0.26, PV^dmPFC^‐IPSC amplitude: *t*_(11)_ = 3.74, *p* = 0.003, PV^dmPFC^‐ frequency E/I ratio: *t*_(11)_ = 3.08, *p* = 0.01, PV^dmPFC^ ‐amplitude E/I ratio: *t*_(11)_ = 2.29, *p* = 0.043; SST^dmPFC^‐EPSC frequency: *t*_(9)_ = 0.18, *p* = 0.86, SST^dmPFC^‐IPSC frequency: *t*_(9)_ = 3.29, *p* = 0.009, SST^dmPFC^‐EPSC amplitude: *t*_(9)_ = 1.23, *p* = 0.25, SST^dmPFC^‐IPSC amplitude: *t*_(9)_ = 1.39, *p* = 0.20, SST^dmPFC^‐ frequency E/I ratio: *t*_(9)_ = 2.57, *p* = 0.03, SST^dmPFC^‐ amplitude: *t*_(9)_ = 0.22, *p* = 0.83, paired Student's *t*‐test). Data are presented as mean ± SEM; ^*^ *p* < 0.05, ^**^ *p* < 0.01, ^***^ *p* < 0.001.

To understand how long‐range vCA1 excitatory inputs are integrated within the dmPFC microcircuit, we combined optogenetic terminal stimulation with cell‐type‐specific fiber photometry. We expressed ChR2 in vCA1 projection neurons and GCaMP6s in either Glu^dmPFC^, PV^dmPFC^ or SST^dmPFC^ (Figure 5A). Phasic optogenetic stimulation of vCA1 terminals elicited profoundly divergent temporal response across these subpopulations. In Glu^dmPFC^, vCA1 terminal activation drove a robust and sustained increase in GCaMP6s fluorescence (Figure 5B,C and Video S1). In striking contrast, inhibitory interneurons displayed highly specialized activation dynamics: SST^dmPFC^ exhibited a transient calcium surge that rapidly decayed to baseline, whereas PV^dmPFC^ showed a complex biphasic response characterized by an initial suppression followed by prolonged excitation (Figure 5D–G and Videos S2 and S3). These distinct responses indicate that hippocampal inputs recruit dmPFC neuronal populations with cell‐type‐specific temporal dynamics.

To delineate the precise anatomical connectivity underlying these functional responses, we employed an anterograde trans‐synaptic tracing strategy using AAV2/1‐Cre in vCA1 and Cre‐dependent EYFP in dmPFC. This mapping confirmed that the vCA1 projection heavily innervates dmPFC, with approximately 80% of targeted postsynaptic cells being glutamatergic and 20% being GABAergic (Figure S6A–C). To functionally validate these connections, we performed targeted whole‐cell patch‐clamp recordings in acute dmPFC slices from specific Cre‐driver mice expressing ChR2 in vCA1 terminals (Figure 5H and Figure S6D). Photostimulation of vCA1 terminals reliably evoked monosynaptic excitatory postsynaptic currents (EPSCs, recorded at −70 mV) as well as inhibitory postsynaptic currents (IPSCs, recorded at 0 mV) across all three identified subpopulations (Figure 5I–K). To determine whether the excitatory responses reflected direct monosynaptic transmission, we performed TTX/4‐AP‐assisted recordings. TTX abolished the light‐evoked EPSCs, whereas subsequent application of 4‐AP restored the EPSCs in dmPFC glutamatergic, PV+, and SST+ neurons (Figure S6E–G), confirming direct monosynaptic excitatory inputs from vCA1 to all three dmPFC populations. In contrast, the IPSCs were not tested with TTX/4‐AP and therefore were interpreted as inhibitory responses recruited by local dmPFC circuits following vCA1 activation.

We next quantified how this long‐range input reshapes E/I balance in the dmPFC microcircuit. In local glutamatergic neurons, vCA1 terminal activation significantly enhanced both the frequency and amplitude of EPSCs and IPSCs; however, the overall E/I ratio remained unchanged, reflecting a proportional “balanced enhancement” of synaptic drive (Figure 5I). In contrast, vCA1 stimulation shifted synaptic dynamics of inhibitory interneurons toward inhibition. PV+ neurons exhibited a significant reduction in E/I ratio, driven by an increase in inhibitory input (Figure 5J). Similarly, in SST neurons, vCA1 input selectively increased IPSC frequency without affecting amplitude, resulting in a decreased frequency‐based E/I ratio (Figure 5K). These findings indicated that vCA1 projections exert cell‐type‐specific synaptic modulation within dmPFC: maintaining balanced excitation and inhibition in glutamatergic neurons, while shifting the synaptic integration of PV+ and SST+ neurons toward a net inhibitory bias (Figure 5I–K and Figure S7).

### 2.5 Selective Manipulation of vCA1–dmPFC Subcircuits Reveals Cell‐Type‐Specific Contributions to Morphine Reward Memory Retrieval

**FIGURE 6:**
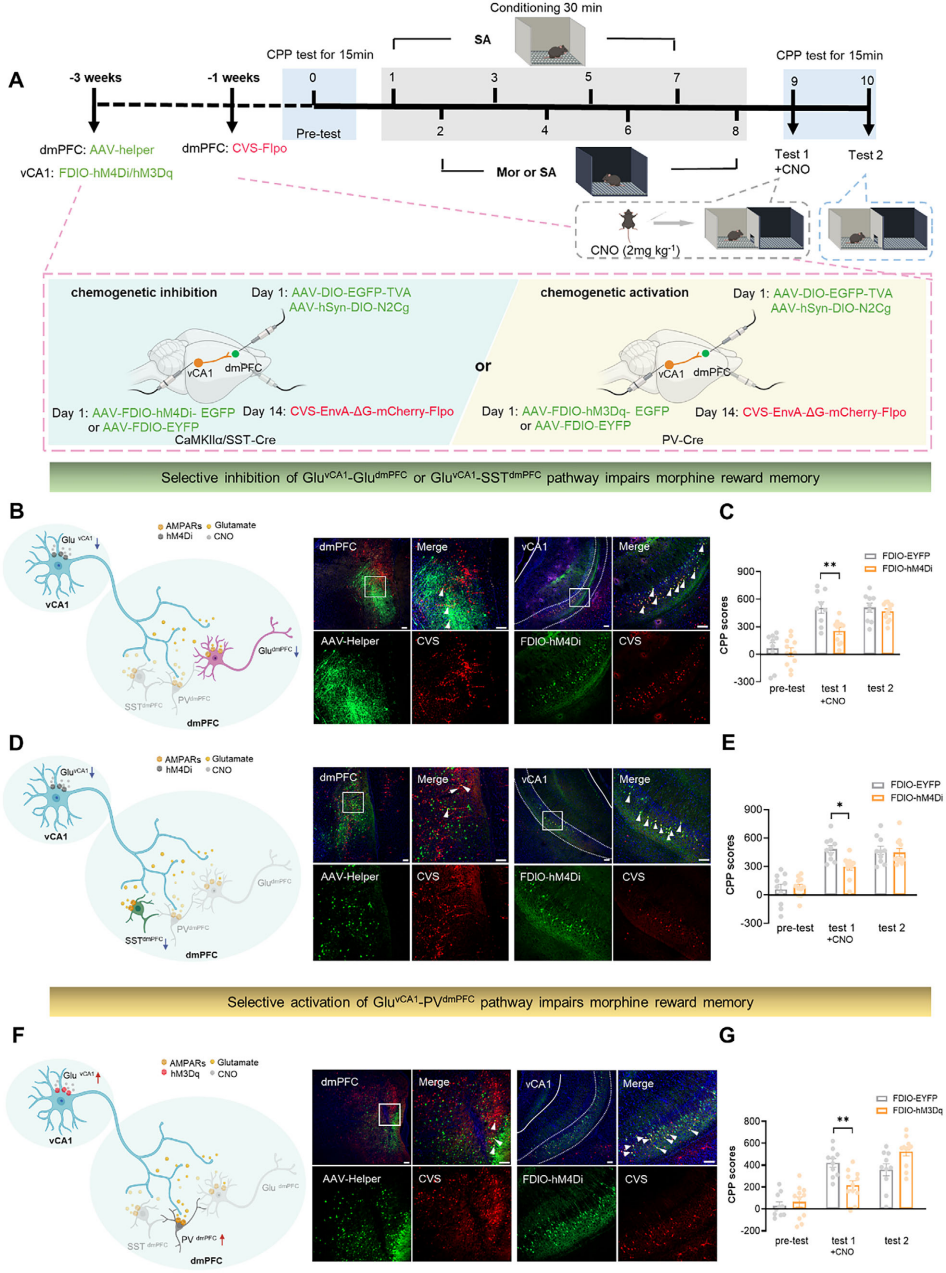
Distinct vCA1–dmPFC neuronal circuits differentially contribute to retrieval of morphine reward memory. (A) Experimental timeline (top) for CPP procedure. Schematic illustration (bottom) of selective manipulation of hippocampal–prefrontal circuits involving Glu^vCA1^ neurons and specific dmPFC neuronal subtypes using intersectional viral strategies. (B, D) Representative fluorescence images showing viral injection sites and expression in dmPFC of CaMKIIα‐Cre and SST‐Cre mice. Starter cells (arrowhead; left) co‐express helper virus rAAV‐DIO‐EGFP‐TVA, rAAV‐DIO‐N2cG (green), and CVS‐EnvA‐ΔG‐mCherry‐P2A‐Flpo (red). Presynaptic neurons in vCA1 labeled by CVS‐mCherry (right) colocalize with FDIO‐hM4Di‐EGFP expression. Scale bars: 100 µm. (C, E) Chemogenetic inhibition of Glu^vCA1^‐Glu^dmPFC^ or Glu^vCA1^‐SST^dmPFC^ pathways via CNO administration significantly reduced CPP scores (C: FDIO‐EYFP group, n = 9; FDIO‐hM4Di group, n = 11; *F*_(2, 36)_ = 2.91, *p* = 0.067; E: FDIO‐EYFP group, n = 10; FDIO‐ hM4Di group, n = 11; *F*_(2, 38)_ = 3.62, *p* = 0.037, two‐way ANOVA). (F) Representative fluorescence images showing viral injection sites and expression in dmPFC of PV‐Cre mice. Starter cells (arrowheads; left) co‐express helper viruses and rabies virus. Co‐labeling of CVS‐mCherry and FDIO‐hM3Dq‐EGFP was observed in vCA1 neurons (right). (G) Chemogenetic activation of PV^vCA1^‐Glu^dmPFC^ projections significantly attenuated CPP scores (FDIO‐EYFP group, n = 10; FDIO‐hM3Dq group, n = 11; *F*_(2, 38)_ = 8.20, *p* = 0.001, two‐way ANOVA). Scale bars: 100 µm. Data are presented as mean ± SEM; ^*^ *p* < 0.05, ^**^ *p* < 0.01.

Our anatomical and electrophysiological data strongly suggest that the divergent physiological responses of PV+ and SST+ neurons are fundamental to their distinct behavioral roles. To test this hypothesis, we employed a highly precise, rabies‐mediated intersectional monosynaptic strategy to selectively manipulate vCA1 inputs projecting onto genetically defined dmPFC subpopulations during reward memory retrieval (Figure 6A). Cre‐dependent helper viruses (rAAV‐DIO‐EGFP‐TVA and rAAV‐DIO‐N2cG) were stereotaxically delivered into the dmPFC of PV‐Cre, SST‐Cre, or CaMKIIα‐Cre mice. Simultaneously, we delivered Flpo‐inducible DREADDs into vCA1. Two weeks later, a modified rabies virus expressing Flpo recombinase (CVS‐EnvA‐ΔG‐mCherry‐P2A‐Flpo) was administered into the dmPFC. This viral strategy ensures that the EnvA‐pseudotyped rabies virus selectively infects Cre+ starter neurons and retrogradely traverses monosynaptically to presynaptic inputs. Consequently, Flpo recombinase is delivered exclusively to vCA1 neurons that directly innervate the targeted dmPFC cells. This intersectional approach allowed us to strictly restrict the expression of excitatory DREADD (hM3Dq‐EGFP) to the Glu^vCA1^‐PV^dmPFC^ pathway, and the inhibitory DREADD (hM4Di‐EYFP) to the Glu^vCA1^‐Glu^dmPFC^ and Glu^vCA1^‐SST^dmPFC^ pathways. Trans‐synaptic specificity was strictly validated by co‐localization of EGFP and mCherry in dmPFC starter neurons and, crucially, by robust co‐expression of mCherry and corresponding chemogenetic constructs within presynaptic vCA1 neurons (Figure 6B,D,F).

Having established selective chemogenetic control over these synaptic connections, we next assessed their roles in morphine memory retrieval. Chemogenetic inhibition of vCA1 inputs targeting either dmPFC glutamatergic neurons or SST+ neurons markedly impaired memory retrieval, resulting in significantly reduced CPP scores (Figure 6C,E). Notably, selective activation of vCA1 inputs onto PV+ neurons similarly disrupted CPP performance (Figure 6G). In contrast, control mice (FDIO‐EYFP) maintained strong preference for the morphine‐paired compartment across all manipulation paradigms. Together, these data demonstrate that distinct vCA1–dmPFC sub‐circuits play dissociable roles in morphine reward memory retrieval: effective retrieval requires excitatory vCA1 input to local pyramidal neurons and SST+ neurons, while excessive recruitment of PV+ neurons disrupts this process.

### 2.6 Cue‐Induced Retrieval of Morphine Reward Memory Requires dmPFC Glutamatergic Signaling

**FIGURE 7:**
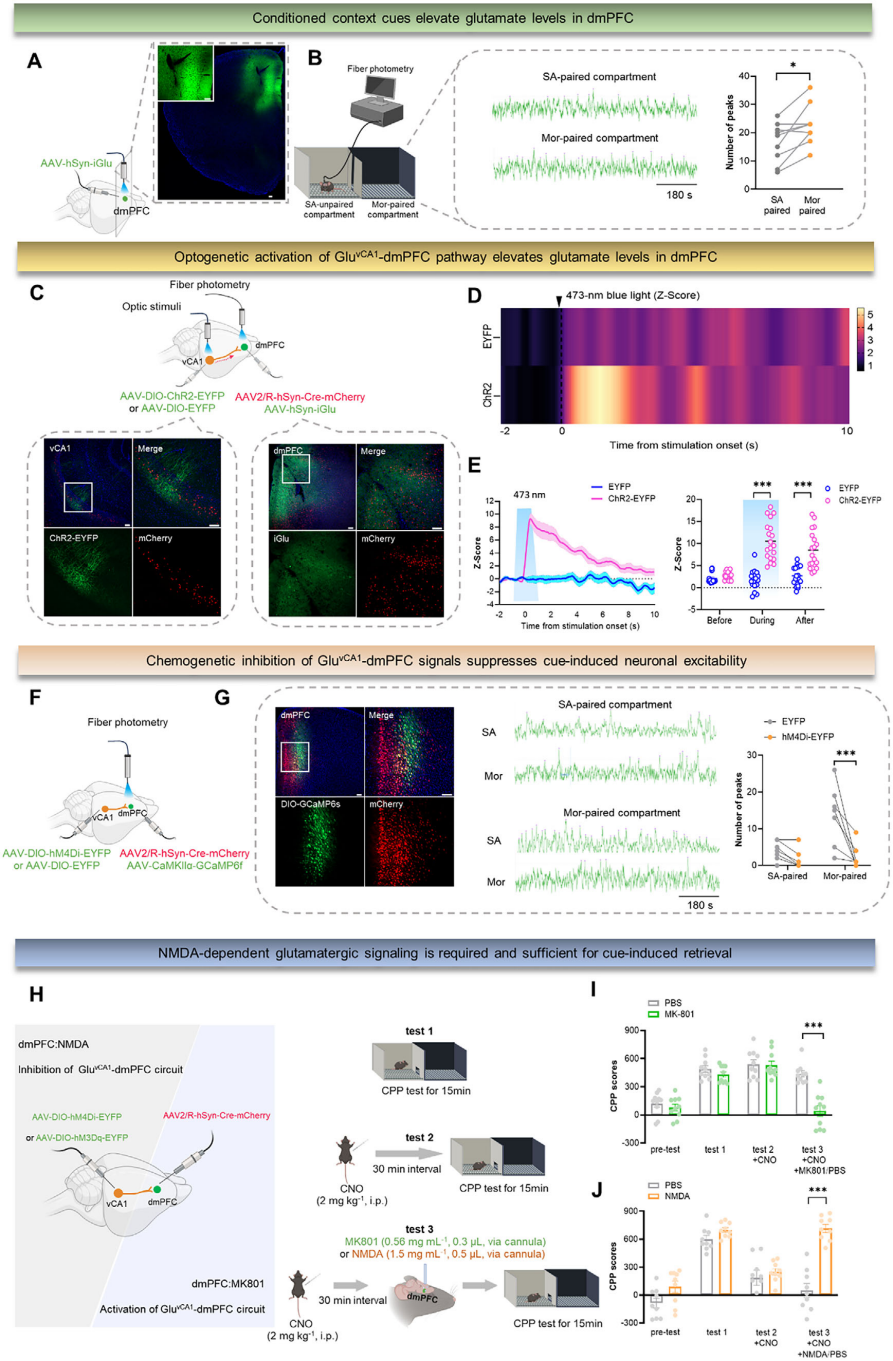
Retrieval of context‐induced morphine reward memory critically depends on glutamatergic signaling in dmPFC. (A) Representative fluorescence images showing injection sites in dmPFC with GRAB_Glu_ sensor expression. Scale bars: 100 µm. (B) Schematic illustration of fiber photometry setup (left) for monitoring glutamate release within dmPFC using GRAB_Glu_ sensors. Representative traces of glutamate signals (middle) and quantification of calcium transient peaks (right) in saline‐ vs. morphine‐paired compartment (n = 9 per group, *t*_(8)_ = 3.25, *p* = 0.012, paired Student's *t*‐test). (C) Schematic design (top) for monitoring dmPFC glutamate dynamics during optogenetic activation of the Glu^vCA1^‐dmPFC pathway via stimulating Glu^vCA1^ cell bodies. Representative images (bottom) showing dmPFC injection sites and co‐localization of mCherry with DIO‐hM4Di‐expressing neurons in vCA1. Scale bars: 100 µm. (D) Heatmaps of GRAB_Glu_ signals aligned to the onset of photostimulation across trials. (E) Representative traces (left) and averaged z‐score (right) of GRAB_Glu_ signals in dmPFC. Bold lines (red for ChR2 and blue for EYFP) indicate the mean; shaded areas represent SEM (EYFP group, n = 18; ChR2 group, n = 19; *F*_(2, 70)_ = 34.18, *p* < 0.001, two‐way ANOVA). (F) Schematic of fiber photometry recording from Glu^dmPFC^ neurons during chemogenetic manipulation via CNO administration. (G) Representative fluorescence images (left) showing viral injection sites and expression in dmPFC. Representative Ca^2+^ transients (middle) and quantification (right) of Glu^dmPFC^ neurons across different conditioning groups (n = 7 per group, *F*_(1, 12)_ = 9.08, *p* = 0.011, two‐way ANOVA). Scale bars: 100 µm. (H) Schematic illustration of pharmacological manipulation experiments involving intra‐dmPFC infusion of MK‐801 or NMDA. (I,J) Local blockade of NMDA receptors with MK‐801 attenuated morphine reward memory retrieval induced by Glu^vCA1^‐dmPFC pathway activation, (PBS control group, n = 9; MK‐801 group, n = 9; *F*_(3, 54)_ = 14.53, *p* < 0.001, two‐way ANOVA), whereas intra‐dmPFC administration of NMDA restored morphine reward memory retrieval impaired by disrupted Glu^vCA1^‐dmPFC signaling (PBS control group, n = 9; NMDA group, n = 9; *F*_(3, 48)_ = 11.84, *p* < 0.001, two‐way ANOVA). Data are presented as mean ± SEM; ^*^ *p* < 0.05, ^***^ *p* < 0.001.

Finally, we examined the real‐time neurotransmitter dynamics underlying this circuit mechanism. To monitor extracellular glutamatergic transmission during memory retrieval, we expressed the genetically encoded glutamate sensor GRAB‐Glu in dmPFC (Figure 7A). Re‐exposure to the morphine‐paired context elicited a robust increase in glutamate transients in dmPFC (Figure 7B).

To determine whether this glutamate signal originated from vCA1 projections, we combined pathway‐specific optogenetic stimulation with GRAB‐Glu imaging. AAV‐DIO‐ChR2‐EYFP was delivered into vCA1, while retroAAV‐Cre and the GRAB‐Glu sensor were co‐injected into the ipsilateral dmPFC (Figure 7C). Optical stimulation of vCA1 terminals in dmPFC produced a rapid and robust increase in GRAB_Glu_ fluorescence, closely mirroring the glutamate dynamics observed during contextual cue exposure (Figure 7D,E and Video S4).

To determine whether vCA1 provides the glutamatergic drive to the dmPFC, we recorded calcium activity from Glu^dmPFC^ expressing GCaMP6f. Chemogenetic inhibition of the vCA1–dmPFC projection markedly reduced cue‐induced calcium transients, indicating a loss of excitatory drive (Figure 7F,G). We next examined the involvement of NMDA receptors by activating the vCA1–dmPFC pathway while locally administering the NMDA receptor antagonist MK‐801 into the dmPFC. Local blockade of NMDA receptors with MK‐801 significantly reduced CPP expression during vCA1–dmPFC pathway activation (Figure 7H,I), supporting a role for dmPFC NMDA receptor signaling in the behavioral effects of this pathway. Conversely, local infusion of NMDA into dmPFC prior to contextual re‐exposure restored morphine CPP expression following pathway inhibition (Figure 7J). Together, these findings established a critical role of dmPFC NMDA receptor signaling in vCA1–dmPFC mediated morphine reward memory retrieval.

### 2.7 Glu^vCA1^ to dmPFC Projections Contribute to Morphine Dependence‐Related Adaptations Without Altering the Acute Analgesic Effect of Morphine

**FIGURE 8:**
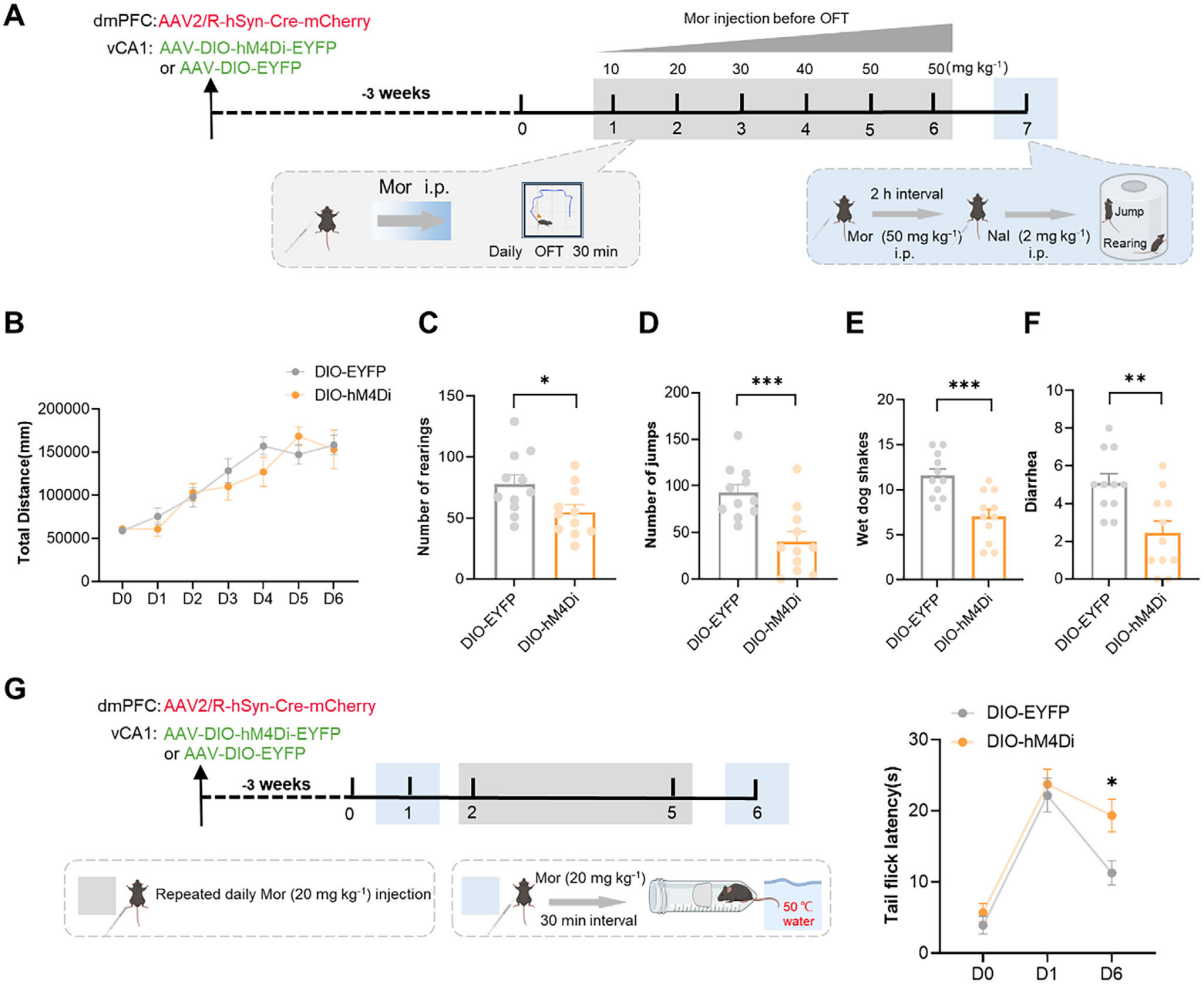
Role of vCA1–dmPFC glutamatergic projections in morphine withdrawal and tolerance‐related behaviors. (A) Experimental timeline for assessing morphine (Mor)‐induced locomotion and naloxone (Nal)‐precipitated withdrawal. (B) Total distance traveled in response to morphine administration under chronic inhibition of Glu^vCA1^‐dmPFC circuit activity (n = 13 per group; *F*_(6, 144)_ = 1.52, *p* = 0.18, two‐way ANOVA). (C‐F) Rearing behavior (C), number of jumps (D), wet‐dog shakes (E) and diarrhea (F) after naloxone administration in mice following chronic inhibition of Glu^vCA1^‐dmPFC circuit during morphine administration (rearing: *t*_(20)_ = 2.33, *p* = 0.030; jump: *t*_(20)_ = 3.87, *p* = 0.001; wet‐dog shakes: *t*_(20)_ = 4.05, *p* < 0.001; diarrhea: *t*_(20)_ = 3.31, *p* = 0.004. n = 11 for per group, unpaired Student's *t*‐test). (G) Experimental timeline for tail flick assay (left) and thermal nociceptive latency across testing days (right) in mice with chronic inhibition of Glu^vCA1^‐dmPFC circuit during morphine administration (n = 13 per group; *F*_(2, 48)_ = 1.61, *p* = 0.21, two‐way ANOVA). Data are presented as mean ± SEM; ^*^ *p* < 0.05, ^***^ *p* < 0.001.

To examine the role of the vCA1–dmPFC pathway in other morphine‐induced behavioral adaptations, we assessed the effects of pathway inhibition on locomotor sensitization, naloxone‐precipitated somatic withdrawal, and analgesic tolerance during chronic morphine exposure. Using an intersectional chemogenetic strategy (retroAAV‐Cre in dmPFC and AAV‐DIO‐hM4Di‐EYFP in vCA1), vCA1 projection neurons were silenced during a 6‐day morphine administration paradigm, with CNO delivered 30 min prior to each daily morphine injection (Figure 8A). Chemogenetic inhibition of the vCA1–dmPFC circuit did not alter morphine‐induced hyperlocomotion (Figure 8B). However, the same manipulation significantly attenuated naloxone‐precipitated somatic withdrawal behaviors, including rearing, jumping, wet‐dog shakes, and diarrhea, on day 7 (Figure 8C–F).

In the tail‐flick test, silencing the vCA1–dmPFC pathway attenuated the development of analgesic tolerance during repeated morphine administration without affecting the acute analgesic response to morphine (Figure 8G). Together, these findings indicate that the vCA1–dmPFC pathway contributes to morphine withdrawal and analgesic tolerance, but is not involved in the acute analgesic effect of morphine.

## 3 Discussion

**FIGURE 9:**
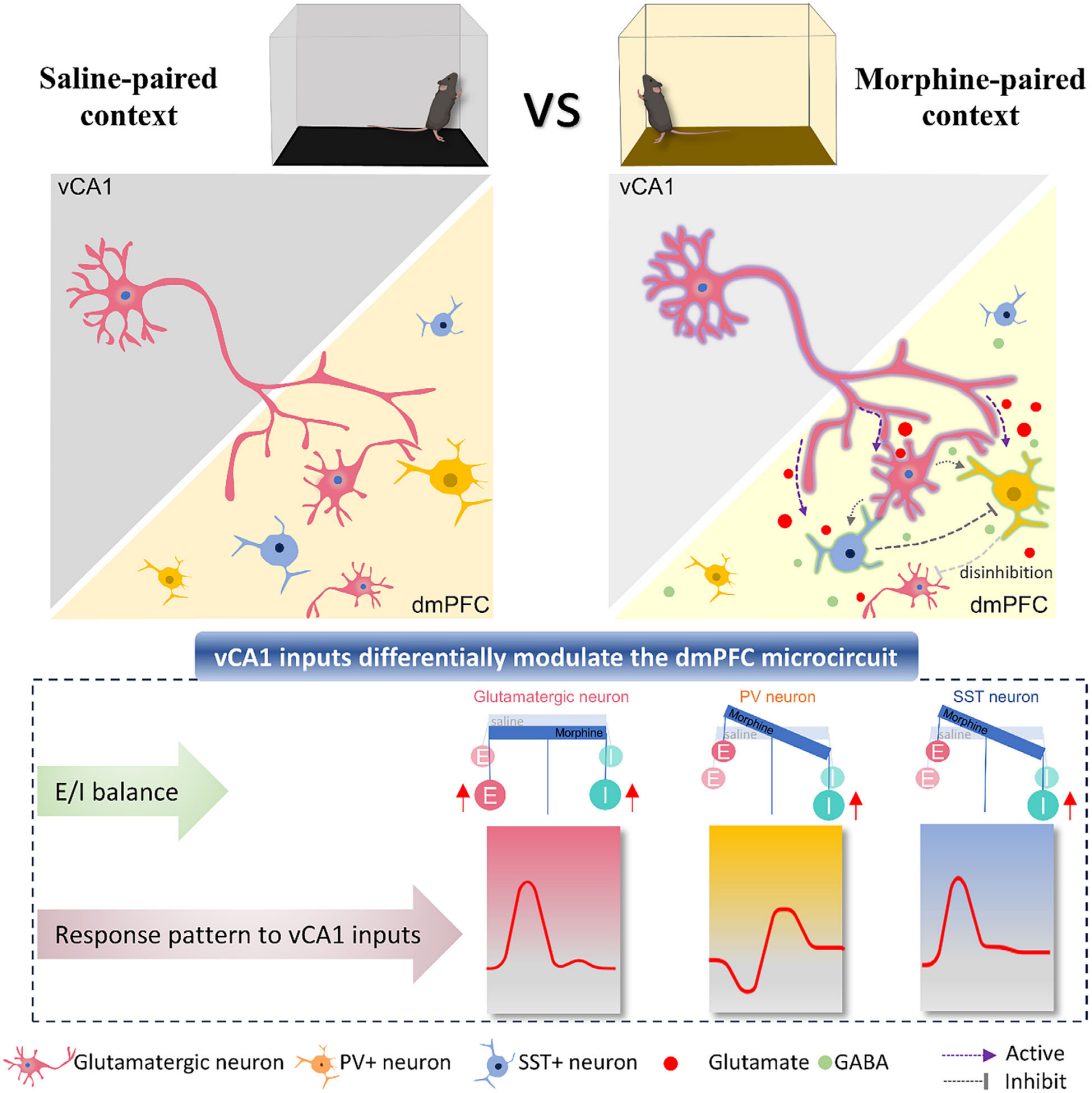
Proposed model of the circuit and molecular mechanism underlying morphine reward memory retrieval. In the saline‐paired context (left), basal vCA1 input maintains a homeostatic excitation/inhibition (E/I) balance across dmPFC glutamatergic neurons and local interneurons (PV+ and SST+). Conversely, re‐exposure to the morphine‐paired context (right) triggers a surge in glutamatergic drive from vCA1. This enhanced input differentially recruits PV+ and SST+ subpopulations, shifting their distinct E/I ratios and engaging feedforward mechanisms. The resulting reconfiguration of interneuron dynamics disinhibits pyramidal neurons, facilitating the output required for morphine reward memory retrieval.

This study identifies a hippocampal–prefrontal cortical circuit in which glutamatergic projections from vCA1 regulate dmPFC microcircuit activity during cue‐triggered retrieval of opioid reward memory (Figure 9). The vCA1–dmPFC pathway also contributes to somatic withdrawal and the development of analgesic tolerance during chronic morphine exposure, while leaving the acute analgesic effect of morphine intact.

The present data show that vCA1 inputs do not simply increase dmPFC excitatory activity but instead engage distinct neuronal populations within the local microcircuit. The hippocampus plays a critical role in the initial formation of long‐term memories. These are gradually consolidated and transferred to the cerebral cortex for storage, where they can be retrieved in response to relevant cues [40, 41, 42, 43, 44, 45, 46]. In drug use disorders, retrieval of drug‐associated memories, particularly those triggered by environmental cues, contributes to craving and relapse [47, 48]. Although efforts have been made to uncover the subcortical circuits underlying reward processing [32, 49, 50, 51], how cortical circuits regulate drug memory retrieval remains less well understood. The dmPFC, a region involved in cognitive flexibility and executive control, receives and integrates inputs from multiple brain regions [32, 52]. Notably, anatomical and functional connectivity between vCA1 and dmPFC is essential for the retrieval of various contextual memories [19, 53]. Cortical information processing depends on interactions between excitatory and inhibitory neurons, and altered GABAergic function has been implicated in addiction [22, 32]. Within the dmPFC, distinct classes of interneurons, particularly PV+ and SST+ neurons, receive excitatory inputs and regulate cortical output [38, 39, 54, 55, 56]. How hippocampal inputs engage these interneuron populations during opioid reward memory retrieval remains unclear.

The present findings suggest that vCA1 inputs regulate opioid memory retrieval through cell‐type‐specific modulation of dmPFC neurons. During morphine memory retrieval, the vCA1–dmPFC projection functions not only as an excitatory input but also regulates local microcircuit activity involving glutamatergic neurons and distinct interneuron populations.

Disruption of this long‐range pathway, either through projection‐specific silencing or local inhibition of dmPFC activity, abolishes memory retrieval, indicating that coordinated hippocampal–prefrontal signaling is required for the expression of morphine‐associated memory. Our circuit mapping revealed direct monosynaptic inputs from vCA1 onto dmPFC PNs as well as PV+ and SST+ neurons. TTX/4‐AP‐assisted electrophysiological experiments further confirmed direct monosynaptic excitatory connections from vCA1 to dmPFC glutamatergic neurons, PV+ interneurons, and SST+ interneurons and demonstrated that hippocampal inputs directly recruit multiple dmPFC cell types. The E/I ratio remained unchanged in glutamatergic neurons, whereas inhibitory input was increased in both PV+ and SST+ interneurons. These optogenetically evoked responses establish the capacity of vCA1 inputs to regulate the dmPFC microcircuit, although they may differ from endogenous activity during natural memory retrieval.

Layer 5 pyramidal neurons are the principal cortical output neurons and receive strong perisomatic inhibition from PV+ interneurons. PV+ interneurons, in turn, can receive rapid and strong excitation from long‐range inputs [57, 58, 59, 60]. In the present study, however, vCA1 stimulation also increased the frequency and amplitude of IPSCs onto PV+ neurons, indicating substantial recruitment of local inhibition. SST+ interneurons provide an additional layer of inhibitory regulation within this circuit. Previous studies have established that SST+ neurons can inhibit PV+ interneurons and thereby regulate the inhibitory control exerted by PV+ cells over cortical output [32, 61, 62]. Consistent with this organization, we found that SST+ neurons directly inhibit PV+ interneurons in the dmPFC. Notably, chronic morphine exposure enhances SST–PV inhibitory transmission, as reflected by increased IPSC amplitudes, indicating plasticity of this local inhibitory connection after repeated morphine exposure. The behavioral data further support a role for dmPFC PV+ and SST+ interneurons in morphine memory retrieval. Activation of PV+ neurons or inhibition of SST+ neurons impaired CPP expression, whereas the opposite manipulations produced no significant effect. Combined with the identification of direct SST–PV inhibitory connections, these findings suggest that SST–PV interactions represent an important component of dmPFC microcircuit regulation during morphine memory retrieval.

The synaptic organization observed ex vivo further illustrates how vCA1 inputs are integrated within the dmPFC microcircuit. Photostimulation of vCA1 terminals evoked monosynaptic EPSCs and polysynaptic IPSCs in dmPFC glutamatergic, PV+, and SST+ neurons. Cortical pyramidal neurons receive feedforward inhibition from PV+ interneurons, whereas PV+ neurons are themselves inhibited by SST+ neurons, forming a well‐established disinhibitory motif. PV+ neurons primarily provide rapid perisomatic inhibition, whereas SST+ neurons preferentially regulate dendritic integration and can inhibit PV+ interneurons [37]. Thus, the cell‐type‐specific synaptic responses observed following vCA1 stimulation are consistent with the distinct roles of these interneuron populations in shaping cortical information processing. Although the full source of inhibitory inputs onto SST+ cells remains to be clarified, potentially involving VIP+ neurons or recurrent inhibitory loops [32], additional local inhibitory pathways are likely to contribute to this regulation.

Beyond the organization of local microcircuits, glutamatergic signaling is a key component of the vCA1–dmPFC pathway during morphine reward memory. Previous studies have shown that chronic morphine administration alone does not impair hippocampal long‐term potentiation (LTP); in contrast, pairing morphine with environmental cues attenuates LTP induction, suggesting an important contribution of contextual associations to morphine‐induced synaptic plasticity [53]. How contextual information is transmitted during memory retrieval, however, remains unclear. Using real‐time GRAB‐Glu imaging, we observed that contextual re‐exposure triggered a robust increase in glutamate release in the dmPFC, and optogenetic activation of vCA1 projections produced a similar increase in dmPFC glutamate signals. Local administration of NMDA into the dmPFC restored memory retrieval following vCA1 pathway inhibition. Conversely, pharmacological blockade of NMDA receptors using MK‐801 impaired morphine CPP retrieval, demonstrating that NMDA receptor‐dependent signaling within the dmPFC is required for the expression of morphine‐associated memory. Together, these complementary gain‐ and loss‐of‐function approaches indicate that NMDA receptor signaling serves as an important downstream mechanism through which vCA1 glutamatergic inputs regulate memory retrieval. Direct activation of dmPFC glutamatergic neurons with hM3Dq, however, failed to restore retrieval after vCA1 inhibition, suggesting that increasing dmPFC glutamatergic neuronal activity alone is insufficient to reproduce the circuit state required for memory expression. Contextual retrieval may instead require glutamate release from vCA1 projections and engagement of local NMDA receptor signaling.

The vCA1–dmPFC pathway also contributes to behavioral adaptations associated with repeated morphine exposure. Chronic inhibition of this circuit not only disrupted reward memory retrieval but also significantly attenuated naloxone‐precipitated somatic withdrawal behaviors (e.g., wet‐dog shakes and jumping) and the development of analgesic tolerance. These findings indicate that the function of the vCA1–dmPFC pathway extends beyond contextual reward memory retrieval and involves broader adaptations associated with opioid dependence and chronic analgesic exposure. Consistent with this interpretation, previous studies have shown that hippocampal–prefrontal projections can regulate opioid tolerance through specific cellular targets, including CCK+ neurons [53]. Our findings extend this work by showing that inhibition of vCA1‐derived glutamatergic input attenuates morphine‐associated memory retrieval, physical withdrawal, and analgesic tolerance, while leaving acute morphine analgesia and baseline cognitive performance unaffected. These findings suggest that the vCA1–dmPFC pathway contributes to several maladaptive adaptations associated with repeated morphine exposure, but is not essential for the acute analgesic effect of morphine.

Together, our findings define a hippocampal–prefrontal mechanism in which contextual information is conveyed through vCA1 glutamatergic projections and integrated by cell‐type‐specific dmPFC microcircuits involving glutamatergic neurons, PV+ and SST+ interneurons to regulate opioid‐associated memory retrieval. Pathway‐specific manipulation, glutamate monitoring, and cell‐type‐resolved electrophysiology demonstrate that vCA1 inputs shape dmPFC circuit activity through selective modulation of local neuronal populations rather than simply increasing cortical excitation. The involvement of this pathway in morphine‐associated memory, withdrawal, and analgesic tolerance, but not acute analgesia, identifies the vCA1–dmPFC pathway as a potential target for reducing opioid‐related maladaptations while preserving therapeutic pain relief.

### 3.1 Limitations of the Study

Several limitations should be considered. First, although our data demonstrate that vCA1 inputs engage SST–PV interactions within the dmPFC microcircuit, the precise temporal dynamics of this inhibitory modulation during natural memory retrieval remain unresolved. Simultaneous monitoring of vCA1 inputs and cell‐type‐specific dmPFC activity will be needed to establish how these interactions evolve during memory retrieval and relate to behavioral output.

Second, although our pharmacological manipulation demonstrates an important role of dmPFC NMDA receptor signaling in morphine memory retrieval, the specific cellular targets remain uncharted. NMDA receptors are expressed in multiple cell populations, including pyramidal neurons, interneurons, and glial cells. Cell‐type‐specific manipulation of NMDA receptors will help define the contribution of respective populations to vCA1‐dependent memory retrieval.

Third, manipulation of dmPFC PV+ and SST+ interneurons that impaired CPP expression was accompanied by a modest reduction in basal locomotor activity. Given the established role of the dmPFC in top‐down motor planning and execution [63, 64], the possible influence of the altered locomotor activity on the behavioral effects observed in the CPP test cannot be completely excluded. Further studies are needed to distinguish the roles of dmPFC microcircuits in locomotor control and reward memory retrieval.

## 4 Materials and Methods

### 4.1 Animals

All animal procedures were approved by the Animal Care and Use Committee of Soochow University (Approval No.: 202206A0770) and were compliant with the Guidelines for the Care and Use of Laboratory Animals (Chinese National Research Council, 2006) and the U.S. National Institutes of Health Guide for the Care and Use of Laboratory Animals. The following mouse strains were used: CaMKIIα‐Cre (JAX stock no. 005359), PV‐Cre (JAX stock no. 012358), SST‐Cre (JAX stock no. 013044) and C57BL/6 (purchased from GemPharmatech Co., Ltd). They were raised in a specific pathogen‐free environment and group housed under a 12 h light/dark cycle (lights on/off at 8:00 am/8:00 pm each day) with food and water available ad libitum. To generate litters, two C57BL/6 females were mated with one male in our facility. The male was removed after 10 days and the females separated into individual cages 1–4 days prior to giving birth. All testing was performed with male mice. Throughout, every effort was made to minimize animal suffering and the number of animals used (Table 1).

**TABLE 1:** Key resource table.

Reagent or resource	Source	Identifier
Antibodies		
Rat Anti‐SST antibody	Millipore	MAB354
Mouse Anti‐PV antibody	Millipore	MAB1572
Rabbit Anti‐CaMKII antibody	Abcam	ab134041
Rabbit Anti‐c‐Fos antibody	CellSignalingTechnology	2250S
Mouse Anti‐DR1 antibody	Santa Cruz	sc‐515083
Alexa fluor 594‐Anti‐Rat secondary antibody	Abcam	ab150
Alexa fluor 488‐Anti‐Rabbit secondary antibody	Invitrogen	A11008
Alexa fluor 488‐Anti‐Mouse secondary antibody	Invitrogen	A11001
Alexa fluor 546‐Anti‐Mouse secondary antibody	Invitrogen	A10036
Bacterial and virus strains		
rAAV‐CaMKIIα‐hM4D(Gi)‐mCherry	BrainCase	BC‐0150
rAAV‐CaMKIIα‐hM3D (Gq) ‐mCherry	BrainCase	BC‐0141
rAAV‐EF1α‐DIO‐hM4D(Gi)‐EYFP	BrainCase	BC‐0154
rAAV‐EF1α‐DIO‐hM3D(Gq)‐EYFP	BrainCase	BC‐0145
rAAV‐ nEF1α‐FDIO‐hM3D(Gq)‐ EGFP	BrainCase	BC‐1048
rAAV‐nEF1α‐FDIO‐hM4D(Gi)‐EGFP	BrainCase	BC‐0158
rAAV2/R‐hSyn‐NLS‐Cre‐P2A‐mCherry	BrainCase	BC‐0539
rAAV2/1‐hSyn‐EGFP‐P2A‐Cre	BrainCase	BC‐0160
rAAV2/R‐hSyn‐mCherry	BrainCase	BC‐0023
rAAV‐EF1α‐DIO‐EGFP‐T2A‐TVA	BrainCase	BC‐0041
rAAV‐hSyn‐DIO‐N2cG	BrainCase	BC‐0304
CVS‐EnvA‐ΔG‐mCherry‐P2A‐Flp0	BrainCase	BC‐RV‐CVS EnvA471
rAAV‐CaMKIIα‐hChR2(H134R)‐EYFP	BrainCase	BC‐0100
rAAV‐CaMKIIα‐eNpHR‐EYFP	BrainCase	BC‐0120
rAAV‐CaMKIIα‐eNpHR‐mCherry	BrainCase	BC‐0121
rAAV‐EF1α‐DIO‐hChR2(H134R)‐EYFP	BrainCase	BC‐0107
rAAV‐CaMKIIα‐GCaMP6f	BrainCase	BC‐0083
rAAV‐EF1α‐DIO‐GCaMP6s	BrainCase	BC‐0086
rAAV‐hSyn‐iGluSnFR3.v857.GPI	BrainCase	BC‐0975
rAAV‐EF1α‐DIO‐EYFP	BrainCase	BC‐0017
rAAV‐EF1α‐FDIO‐EYFP	BrainCase	BC‐0015
rAAV‐CaMKIIα‐mCherry	BrainCase	BC‐0028
rAAV‐fSST‐flpo‐polyA	BrainVTA	PT‐8972
rAAV‐EFla‐FDIO‐hChR2(H134R)‐mCherry	BrainVTA	PT‐1874
Chemicals, peptides and recombinant proteins		
Clozapine‐N‐oxide (CNO)	MedChemExpress	HY‐17366
Dizocilpine maleate (MK‐801)	MedChemExpress	HY‐15084
4‐Aminopyridine	Sigma‐Aldrich	275875
N‐Methyl‐D‐aspartic acid hydrate (NMDA)	Sigma‐Aldrich	454575
Naloxone hydrochloride	Tocris	0599
Tetrodotoxin	Chengdu Institute of Biology	A0224
Morphine	Shenyang First Pharmaceutical Factory	

### 4.2 Stereotaxic Surgery and Virus Construction

Mice were anesthetized with sodium pentobarbital (60 mg kg^−1^, i.p.) and placed in a stereotaxic apparatus (RWD Instruments, China). Sterile ophthalmic ointment was applied to protect the eyes, and body temperature was maintained at 37°C using a heating pad. After disinfection and a midline scalp incision, the skull surface was exposed and levelled. Cranial windows were opened using a high‐speed drill (RWD Instruments, China) to allow for virus injection, cannula implantation, or optical fiber placement. For viral delivery, 100–400 nL of virus was injected into the dorsomedial prefrontal cortex (dmPFC: AP +1.9 mm, ML ± 0.3 mm, DV −2.0 mm) or ventral CA1 (vCA1: AP −3.3 mm, ML ± 3.3 mm, DV −4.0 mm) at a rate of 30 nL min^−1^ using a calibrated glass micropipette connected to a micro pump (Micro4, World Precision Instruments, USA). The pipette was left in place for 10 min post‐injection to allow for viral diffusion before withdrawal. The incision was sutured, and animals were allowed to recover on a 37°C heating pad. Three weeks post‐injection, when required, guide cannulas (RWD Instruments, China) or optical fibers (Inper Technology, China) were stereotactically implanted into dmPFC or vCA1. Implants were secured with skull screws and dental acrylic.

For chemogenetic experiments, 300–400 nL of rAAV‐CaMKIIα‐hM4D(Gi)‐mCherry (AAV2/9, 3.01 × 10^12^ vg mL^−1^) or rAAV‐CaMKIIα‐hM3D(Gq)‐mCherry (AAV2/9, 3.05 × 10^12^ vg mL^−1^) was injected into dmPFC or vCA1 of mice to manipulate glutamatergic neuronal activity in target regions.

To achieve pathway‐specific chemogenetic inhibition of the vCA1‐dmPFC circuit, two strategies were employed: 1. 400 nL of rAAV‐CaMKIIα‐hM4D(Gi)‐mCherry (AAV2/9, 3.01 × 10^12^ vg mL^−1^) was injected into vCA1 of mice. Three weeks later, a guide cannula (C = 3 mm, G1 = 0.5 mm, G2 = 0.5 mm) was implanted above the dmPFC and secured with dental cement. 0.2 µL of 3 µM CNO was locally infused into dmPFC using a microinfusion pump 30 min before the experiment; 2. 200 nL of rAAV‐hSyn‐NLS‐Cre‐P2A‐mCherry (AAV2/R, 5.67 × 10^12^ vg mL^−1^) was injected into dmPFC, and 300 nL of rAAV‐EF1α‐DIO‐hM4D(Gi)‐EYFP (AAV2/9, 3.01 × 10^12^ vg mL^−1^) into vCA1.

To manipulate specific neuronal subtypes in dmPFC, 300 nL of rAAV‐EF1α‐DIO‐hM3D(Gq)‐EYFP (AAV2/9, 2.0 × 10^12^ vg mL^−1^) or rAAV‐EF1α‐DIO‐hM4D(Gi)‐EYFP (AAV2/9, 3.01 × 10^12^ vg mL^−1^) was injected into the dmPFC of (PV‐Cre or SST‐Cre) mice.

For selective manipulation of Glu^vCA1^‐Glu^dmPFC^, Glu^vCA1^‐SST^dmPFC^, and Glu^vCA1^‐PV^dmPFC^ circuits, an engineered CVS‐N2c‐based trans‐monosynaptic retrograde strategy was applied. Two viral injections were performed in transgenic mice (PV‐Cre, SST‐Cre, or CaMKIIα‐Cre). In the first step, a mixture of helper AAVs (rAAV‐EF1α‐DIO‐EGFP‐T2A‐TVA and rAAV‐hSyn‐DIO‐N2cG; volume ratio 1:2; total 150–200 nL; AAV2/9, 3.0 × 10^12^ vg mL^−1^) was injected into dmPFC. Simultaneously, 400 nL of rAAV‐nEF1α‐FDIO‐hM3D(Gq)‐EGFP (AAV2/9, 2.0 × 10^12^ vg mL^−1^) or rAAV‐nEF1α‐FDIO‐hM4D(Gi)‐EYFP (AAV2/9, 3.6 × 10^12^ vg mL^−1^) was injected into vCA1. After 2–3 weeks, 200 nL of CVS‐EnvA‐ΔG‐mCherry‐P2A‐Flp0 (CVS, 5.0 × 10^8^ IFU mL^−1^) was injected into the same dmPFC site. Behavioral testing was conducted 7–10 days later.

For optogenetic experiments, 300–400 nL of rAAV‐CaMKIIα‐hChR2(H134R)‐EYFP (AAV2/9, 5.66 × 10^12^ vg mL^−1^) or rAAV‐CaMKIIα‐eNpHR3.0‐mCherry (AAV2/9, 2.0 × 10^12^ vg mL^−1^) was injected into dmPFC or vCA1 to modulate neuronal activity. For pathway‐specific optogenetic manipulation of the vCA1‐dmPFC projection, 200 nL of rAAV‐hSyn‐NLS‐Cre‐P2A‐mCherry (AAV2/R, 5.67 × 10^12^ vg mL^−1^) was injected into dmPFC and 300 nL of rAAV‐EF1α‐DIO‐hChR2‐EYFP (AAV2/9, 5.06 × 10^12^ vg mL^−1^) into vCA1. A ceramic ferrule (200 µm O.D., 0.39 NA; RWD, China) was implanted 200 µm above the viral injection site and fixed in place with dental cement.

For anterograde tracing, 300 nL of rAAV‐hSyn‐EGFP‐P2A‐Cre (AAV2/1, 1.03 × 10^13^ vg mL^−1^) was injected into vCA1 and 200 nL of rAAV‐EF1α‐DIO‐EYFP into dmPFC. Cre recombinase expressed in vCA1‐projecting axons triggered EYFP expression in downstream dmPFC neurons. For retrograde tracing, 250 nL of AAV/Retro‐hSyn‐mCherry (AAV2/R, 5.07 × 10^12^ vg mL^−1^) was injected into dmPFC to label upstream vCA1 neurons.

Control injections included rAAV‐CaMKIIα‐mCherry (AAV2/9, 2.0 × 10^12^ vg mL^−1^) rAAV‐EF1α‐DIO‐EYFP (AAV2/9, 2.0 × 10^12^ vg mL^−1^) and rAAV‐EF1α‐FDIO‐EYFP (AAV2/9, 2.0 × 10^12^ vg mL^−1^).

### 4.3 Optogenetic and Chemogenetic Manipulation In Vivo

After three weeks of virus expression, mice were transported to a testing room and were habituated for approximately 1 h. Mice were then anesthetized with isoflurane to connect the chronically implanted fibers to a laser generator using optic fiber sleeves and returned to the home cage for at least 30 min. Next, optogenetic stimulation experiments were carried out using blue light (473 nm, 5–8 mW, 15 ms pulses, 20 Hz) or continuous yellow light (594 nm, 5–8 mW, constant) controlled via the built‐in digital‐to‐analog converter (DAC) of the CoolLED system (CoolLED, UK). For chemogenetic manipulation, clozapine‐N‐oxide (CNO, 2 mg kg^−1^, i.p.) was administered 30 min prior to behavioral testing. For the control group, the same stimulus protocol was performed. After all behavioral tests were completed, mice were killed to verify the virus injection site and the optic fiber site. Data obtained from mice in which the injection site was outside the targeted brain regions were excluded from analysis.

### 4.4 Cannula Implantation and Microinjection

Mice were anesthetized with sodium pentobarbital (60 mg kg^−1^, i.p.). After scraping away the pericranium, burr holes were made on the skull. Guide cannulae were implanted bilaterally above the dmPFC (AP: +1.9 mm; ML: ± 0.3 mm; DV: −1.50 mm). Cannulae were anchored to the skull with stainless‐steel screws and dental cement. Microinjections were made through a needle connected to a 10 µL microsyringe (Gaoge Micro Injector, Shanghai) mounted on a syringe pump (RWD Instruments, China). The needle extended 500 µm beyond the tip of the guide cannula to prevent blockage. For activation of the NMDA receptor, a total volume of 0.5 µl NMDA (1.5 mg mL^−1^ in PBS, 15 min before CPP procedures, given once) was injected into the dmPFC bilaterally via implanted cannulae. For inhibition of NMDA receptor signaling, MK‐801 (0.56 mg mL^−1^, 0.3 µL per side, 15 min before CPP procedures, given once) was infused into dmPFC via implanted cannulae. For the inhibition or activation of the vCA1‐dmPFC circuit, 0.2 µL CNO (3 µM; 30 min before optogenetic procedures, given once) was injected into dmPFC. The infusion rate of all drugs was 0.1 µL min^−1^. After infusion, the needle was left in place for another 3 min to allow drug diffusion. Data obtained from mice in which the injection site was outside the targeted brain regions were excluded from analysis.

### 4.5 Fiber Photometry

Calcium signals were recorded using fiber photometry as previously described [65, 66]. For calcium signals of Glu^vCA1^ and Glu^dmPFC^, 300 nL of rAAV‐CaMKIIα‐GCaMP6f was injected into dmPFC and vCA1, and the optical fiber was implanted above each injection site. To detect the calcium signals of PV^dmPFC^ and SST^dmPFC^ neurons, rAAV‐EF1α‐DIO‐GCaMP6s was injected into dmPFC, and an optical fiber was implanted above the dmPFC of PV‐Cre or SST‐Cre mice. To confirm the calcium signals of Glu^dmPFC^, PV^dmPFC^, and SST^dmPFC^ neurons when Glu^vCA1^ neurons were optically activated, AAV‐CaMKIIa‐ChR2‐mCherry virus was injected into vCA1, AAV‐DIO‐GCaMP6s virus was injected into dmPFC, and an optical fiber was implanted above the dmPFC of CaMKIIa‐Cre, PV‐Cre, or SST‐Cre mice. To examine glutamate release, 300 nL of rAAV‐hSyn‐iGluSnFR3.v857GPI was injected into the dmPFC. To evaluate vCA1‐evoked glutamate transmission, 250 nL of retro‐AAV‐hSyn‐NLS‐Cre‐P2A‐mCherry was co‐injected with the glutamate sensor virus into dmPFC, and 400 nL of rAAV‐EF1α‐DIO‐hChR2(H134R)‐EYFP was injected into vCA1.The fiber photometry recording was conducted after three weeks of viral expression, and a mono fiber optical patch cord (RWD, R‐FC‐L‐N3‐200‐1.1) connected to the fiber photometry system (RWD) was attached to the implanted fiber optical cannula using a ceramic sleeve with black heat‐shrinkable tubes. Before recording, mice were allowed to rest and habituate for 3 min. 473 and 410 nm lasers were used for GCaMP6f or DIO‐GCaMP6s signal and autofluorescence measurement. The 410 nm channel served as the control channel and was subtracted from the GCaMP6s channel to eliminate autofluorescence, bleaching, and motion effects. Light at the fiber tip ranged from 10 to 20 µW (410 nm) and 20–40 µW (473 nm) and was constant across trials over testing days. Change in fluorescence during optical stimulation (−2 to 10 s) or the total CPP test phase (0–900 s) session was calculated and analyzed by analysis software developed by RWD. F0 was defined as the mean baseline signal from −2 s to 0 s for single optical stimulation or 0–900 s for the total CPP test. All heatmaps and averaged calcium traces with shaded areas denoting SEM were generated in the analysis software developed by RWD. Following recordings, mice were perfused transcardially with 4% paraformaldehyde (PFA) and processed to confirm viral expression and placement of optical fibers. Data obtained from mice in which viruses and fibers were located outside the targeted brain regions were excluded from analysis.

### 4.6 Acute Brain Slice Preparation

Mice were anesthetized with sodium pentobarbital (60 mg kg^−1^, i.p.) and perfused transcardially with ice‐cold N‐methyl‐D‐glucamine (NMDG)‐based cutting solution containing (in mM): 93 NMDG, 2.5 KCl, 1.2 NaH_2_PO_4_, 10 MgSO_4_, 0.5 CaCl_2_, 20 HEPES, 3 sodium pyruvate, 30 NaHCO_3_, and 25 D‐glucose (pH adjusted to 7.4 with 10‐N HCl; 300–310 mOsm), continuously bubbled with 95% O_2_/5% CO_2_. Coronal brain slices (330 µm thick) containing the dmPFC or vCA1 were cut using a vibratome (VT1000S, Leica Microsystems, Nussloch, Germany) in the same ice‐cold dissection solution. Following sectioning, slices were first incubated in NMDG solution at 32°C for 6 min, then transferred to a holding chamber containing artificial cerebrospinal fluid (aCSF) composed of (in mM): 125 NaCl, 2.5 KCl, 1.3 NaH_2_PO_4_, 1.3 MgCl_2_·6H_2_O, 2 CaCl_2_·2H_2_O, 25 NaHCO_3_, and 20 D‐glucose, equilibrated with 95% O_2_/5% CO_2_ at 28°C for at least 60 min before electrophysiological recordings. All slices were used within 6 h of preparation.

### 4.7 Whole‐Cell Recordings

#### 4.7.1 Slice Electrophysiology

Brain slices were transferred to a submersion‐type recording chamber and continuously perfused with artificial cerebrospinal fluid (ACSF), bubbled with 95% O_2_ and 5% CO_2_, and maintained at 28°C. Slices were visualized under an upright microscope (FN1, Nikon, Japan) equipped with differential interference contrast (DIC) optics and an infrared CCD camera (IR‐1000E, DAGE‐MTI, Michigan, IN, USA). Whole‐cell patch‐clamp recordings were performed on neurons in dmPFC or vCA1 using a MultiClamp 700B amplifier (Molecular Devices, Sunnyvale, CA, USA). Signals were low‐pass filtered at 2 kHz and digitized at 10 kHz using a Digidata 1550B system (Molecular Devices).

For current‐clamp recordings, borosilicate glass pipettes (resistance: 4–6 MΩ) were filled with a potassium‐based internal solution containing (in mM): 110 K‐gluconate, 20 HEPES, 20 KCl, 5 MgCl_2_, 0.6 EGTA, 2 MgATP, and 0.2 Na_3_GTP (pH 7.3, 290 mOsm). Liquid junction potential and series resistance were not compensated. A small bias current was applied to maintain the resting membrane potential at −60 mV for dmPFC pyramidal or vCA1 glutamatergic neurons, and −40 mV for PV+ or SST+ interneurons in dmPFC. Neurons were considered silent if their average firing rate was <0.05 Hz. Both male and female animals of all genotypes were used in the study.

To examine neuronal excitability in vCA1 following 8 days of daily intraperitoneal (i.p.) injection of morphine or saline, only vCA1 neurons that were retrogradely labeled from dmPFC were recorded. For spike frequency vs. injected current analysis, neurons were subjected to 500 ms depolarizing current injections, and the average action potential frequency was quantified.

#### 4.7.2 vCA1‐dmPFC Circuit Manipulation Ex Vivo

To assess functional connectivity and optogenetic manipulation of the vCA1–dmPFC pathway, rAAV‐CaMKIIα‐hChR2(H134R)‐EYFP was injected into vCA1 of CaMKIIα‐Cre, PV‐Cre, or SST‐Cre mice, and rAAV‐EF1α‐DIO‐EGFP was injected into dmPFC. Blue light (475 nm) was delivered via a light‐emitting diode (LED; pE‐300 ultra, CoolLED, UK) coupled to a 40× objective (NA = 0.8, Nikon FN1), controlled by pClamp software, to activate ChR2‐expressing cells or axon terminals. Evoked spikes in fluorescently labeled vCA1 or dmPFC neurons were recorded in response to blue light pulses (5, 10, or 20 Hz; pulse width: 20 ms; inter‐pulse interval: 200 ms). For synaptic connectivity mapping of the vCA1–dmPFC pathway, light‐evoked synaptic responses were further validated using a TTX/4‐AP protocol. Tetrodotoxin (TTX, 1 µM) was applied to block action potential‐dependent transmission, followed by addition of 4‐aminopyridine (4‐AP, 1 mM) to restore axonal terminal depolarization. Restoration of light‐evoked EPSCs after TTX/4‐AP application was used to confirm monosynaptic excitatory connections from vCA1 terminals onto dmPFC glutamatergic neurons, PV+ interneurons, and SST+ interneurons.

For optogenetic inhibition experiments, rAAV‐CaMKIIα‐NpHR3.0‐mCherry was expressed in vCA1 neurons. Yellow light (589 nm) was continuously applied during depolarizing current injections to assess hyperpolarization‐induced suppression of spiking.

For chemogenetic experiments, spike frequencies were recorded before and after bath application of clozapine‐N‐oxide (CNO, 10 µM). All recordings were analyzed using Clampfit 11.0.3 software.

### 4.8 Synaptic Transmission and E/I Ratio

To investigate synaptic connectivity between vCA1 and dmPFC, light‐evoked excitatory (eEPSCs) and inhibitory (eIPSCs) postsynaptic currents were recorded in dmPFC neurons held at −70 and 0 mV, respectively, following 20 ms blue light stimulation of ChR2‐expressing vCA1 terminals, as previously described [67].

For E/I ratio measurements, a cesium‐based low‐chloride internal solution was used, containing (in mM): 130 CsMeSO_3_, 1 MgCl_2_, 1 CaCl_2_, 10 HEPES, 2 QX‐314, 11 EGTA, 2 MgATP, and 0.3 Na_3_GTP (pH 7.3, 290 mOsm). Cells were voltage‐clamped at −70 mV to record eEPSCs and then at 0 mV to record eIPSCs. Series and input resistances were monitored every 10 s with a 100 ms, −10 mV test pulse. Cells with input resistance changes >20% were excluded. Only recordings with series resistance <20 MΩ were included. No compensation was applied for junction potentials or series resistance. MiniAnalysis software (Synaptosoft, Fort Lee, NJ, USA) was used to detect eEPSCs and eIPSCs with thresholds of 5 and 6 pA, respectively. All analyses were conducted blind to experimental conditions.

### 4.9 SST–PV Synaptic Connectivity

To examine inhibitory connectivity between SST+ and PV+ interneurons in dmPFC, rAAV‐fSST‐flp and rAAV‐FDIO‐hChR2‐mCherry were co‐injected into the dmPFC of PV‐Cre mice to selectively express ChR2 in SST+ interneurons, while PV+ interneurons were identified based on Cre‐dependent labeling. Blue light stimulation (475 nm, 20 ms) was applied to activate SST+ interneurons, and light‐evoked inhibitory postsynaptic currents (IPSCs) were recorded from PV+ interneurons at a holding potential of 0 mV. To assess morphine‐induced plasticity of SST–PV connectivity, recordings were performed in mice after repeated morphine or saline administration, and IPSC amplitudes were compared between groups.

### 4.10 Immunohistochemistry

Mice subjected to behavioral analysis were deeply anesthetized and perfused transcardially with saline followed by 4% PFA in 0.1 M PBS. Brains were removed, post‐fixed overnight in 4% PFA at 4°C, and transferred into 30% sucrose in 0.1 M PBS for dehydration. Coronal sections (30 µm) were cut on a cryostat (Leica, Germany) and stored in 0.1 M PBS. The sections were incubated in blocking buffer containing 1% bovine serum albumin (BSA) and 3% normal goat serum in 0.5% TritonX‐ 100/PBS for 1.5 h at room temperature and then with primary antibodies in blocking buffer overnight at 4°C. The primary antibodies used were: c‐fos (1:1000, CST), PV (1:500, Millipore), SST (1:500, Millipore), and CaMKII (1:400, Millipore). After three washes with PBS, sections were incubated with either Alexa Fluor 488‐ or Alexa Fluor 594‐conjugated secondary antibodies at room temperature for 2 h. After another three washes in PBS, sections were incubated with DAPI (Beyotime, C1005) for 30 min and then washed three times. After rinsing, the sections were mounted, and confocal images were captured with a Nikon A1 microscope (Tokyo, Japan).

### 4.11 Behavioral Procedures

All behavioral tests were conducted during the light cycle after mice had been handled for at least three days. Prior to testing, mice were habituated to the behavioral room for at least one hour. The behavior apparatus was thoroughly cleaned with 70% ethanol after each trial to eliminate any olfactory cues.

#### 4.11.1 Open Field Test

The open field test (OFT) was used to measure locomotor activity. Briefly, animals were habituated in the experimental room 30 min prior to the experiment. Mice were then placed in the center of the OFT apparatus (20 cm length × 20 cm width × 40 cm height) and allowed to explore the area for 30 min. Locomotion trials were recorded using a camera directly above the apparatus, and total distance was measured using a behavior analysis system (VisuTrack system, Shanghai, China) as previously described [16].

#### 4.11.2 Novel Object Recognition Test

For the novel object recognition (NOR) test, one day before testing, mice were placed individually in the test chamber to explore freely for 15 min. The next day, two objects of the same shape and color were placed in opposite positions on the diagonal of the test chamber. During the training phase, mice were placed in the center of the test chamber with their heads facing in the same direction and allowed to explore freely for 5 min and then removed. After an interval of 10 min, one of the objects was replaced with a novel object of a different color and shape. In the recognition phase, the mice were placed in the center of the test chamber with their heads facing in the same direction and allowed to explore freely for 5 min. Video acquisition software was used to record exploration by mice for 5 min, and the time spent exploring the novel object and the familiar object was counted manually. Effective exploration was indicated by sniffing behavior; leaning on or crossing the object was not included in exploration time.

#### 4.11.3 Conditioned Place Preference

The CPP apparatus (Jiliang Ltd., Shanghai, China) contains two distinct visual and textural cue compartments: a dark plexiglass/polyvinyl chloride box with fine wire mesh floor and a white plexiglass/polyvinyl chloride box with wide grid floor. A removable gate between the two boxes ensured that mice were free or restricted to cross the apparatus during different experimental periods. Time spent in each box was recorded and analyzed through infrared beams and an automated analysis system for baseline and test periods, as previously described [16].

The CPP protocol consisted of three phases:

- Habituation phase (Day 0): Mice were placed in the apparatus with the partition removed and allowed to freely explore both compartments for 15 min. Locomotor activity and time spent in each chamber were recorded using behavioral tracking software. Animals that exhibited a strong unconditioned preference (>65% of time, i.e.,>585 s, in one compartment) were excluded from subsequent analysis.
- Conditioning phase (Days 1–8): During this phase, the partition was inserted to restrict animals to a single compartment. Mice received alternating treatments during the same time window each day. On odd‐numbered days (days 1, 3, 5, and 7), mice received an intraperitoneal (i.p.) injection of saline and were confined to the non‐drug‐paired compartment for 30 min. On even‐numbered days (days 2, 4, 6, and 8), mice received an i.p. injection of morphine (20 mg kg^−1^) and were confined to the drug‐paired compartment for 30 min. The apparatus was cleaned between sessions with odor remover and tissues to eliminate olfactory cues.
- Test phase (Days 9–10): On test day 1, the partition was removed to allow free exploration of both compartments for 15 min; for chemogenetic experiments, mice received an injection of CNO (2 mg kg^−1^, i.p.) 30 min prior to testing; for optogenetic experiments, light stimulation was delivered according to the experimental protocol while animals were allowed to explore freely.

On test day 2, mice underwent the same procedure but received saline (chemogenetics) or control‐wavelength light (optogenetics) instead of active treatment.

#### 4.11.4 Real‐Time Place Preference (RTPP) Test

The RTPP test evaluates the immediate reinforcing or aversive effects of neural manipulation using a two‐phase protocol:

Baseline phase: Mice were placed in the two‐chamber apparatus with the partition removed and allowed to explore freely for 15 min. Time spent in each chamber was recorded. Animals that spent more than 65% of the total time (>585 s) in either chamber were excluded from further testing.

Test phase: One compartment was randomly assigned as the stimulation chamber. Animals were allowed to explore both chambers freely. When the center of mass of the animal entered the stimulation chamber, a signal was sent from the tracking software to the optogenetic stimulation system (e.g., LED or laser) to trigger light delivery in real time. Time spent in the stimulation chamber was automatically recorded for quantification.

Preference score was calculated as time spent in the drug‐paired chamber − time spent in the non‐drug‐paired chamber.

#### 4.11.5 Morphine‐Induced Locomotion and Naloxone‐Precipitated Withdrawal Test

Morphine‐induced locomotor activity was assessed using the same apparatus as the open field test. On Day 0, mice were placed in the chamber and allowed to freely explore for 30 min to establish baseline locomotion. From Days 1–6, mice were habituated to the apparatus to reduce novelty‐driven activity.

To evaluate the effect of pathway inhibition on morphine‐induced hyperlocomotion, mice received CNO (2 mg kg^−1^, i.p.) 30 min prior to morphine administration. Morphine (20 mg kg^−1^, i.p.) was then administered, and locomotor activity was recorded for 30 min. Total distance was measured using a behavior analysis system (VisuTrack system, Shanghai, China).

To determine whether pathway inhibition altered naloxone‐precipitated withdrawal, mice received CNO (2 mg kg^−1^, i.p.) 30 min prior to morphine administration. Morphine was then administered at doses of 10, 20, 30, 40, or 50 mg kg^−1^ (i.p.), and locomotor activity was recorded for 30 min. On Day 7, naloxone‐precipitated withdrawal symptoms were assessed. Mice received 50 mg kg^−1^ morphine (i.p.) and remained in their home cages. Two hours later, naloxone (2 mg kg^−1^, i.p.) was administered to induce withdrawal. Behavior was recorded for 20 min using a digital video camera. Withdrawal symptoms, including the number of jumps and front paw lifts, were quantified by an observer blind to treatment conditions.

#### 4.11.6 Tail Flick Test

The tail flick test was performed to assess the analgesic effects of morphine and the development of tolerance upon repeated administration. Mice were gently restrained in 50 mL centrifuge tubes with the tail extending through a hole at the tip. After a 5–10 min habituation, the distal 2–3 cm of the tail was immersed in a 50°C water bath. Tail withdrawal latency was recorded as the time to flick in response to the thermal stimulus.

To prevent tissue damage, a 30 s cut‐off was applied. Baseline latencies were measured on day 0. Mice then received 20 mg kg^−1^ morphine (i.p.) once daily for six consecutive days. Tail flick latencies were assessed on days 1 and 6, 40 min after morphine injection, to evaluate analgesic response and the development of tolerance.

### 4.12 Statistics

Statistical analysis and graphics were performed using GraphPad Prism 9.0 (GraphPad Software). Data are presented as means ± standard errors of the mean (SEM). All molecular biological and biochemical assays were repeated at least three times as noted in figure legends. Differences between two groups were determined using Student's *t*‐test, with one‐way or two‐way ANOVA used to analyze more than two groups, followed by Tukey's post hoc test. Significance levels are indicated as ^*^ *p* < 0.05, ^**^ *p* < 0.01, ^***^ *p* < 0.001.

## Author Contributions

Z.‐F.S. performed experiments, analyzed the data, prepared the figures, and wrote the manuscript. G.‐Y.L. and Q.Y. performed electrophysiological experiments and analyzed the corresponding data. R.‐S.L. performed immunohistochemistry and analyzed the data. L.‐H.G. and M.‐M.W. contributed to data collection. J.L.W. and F.‐H.J. analyzed and interpreted the data and revised the manuscript. X.‐C.Z. conceived and supervised the study, designed the experiments, and finalized the manuscript. All authors reviewed and approved the final manuscript.

## Conflicts of Interest

The authors declare no conflicts of interest.

## Data and Materials Availability

The data that supports the findings of this study are available in the supplementary material of this article.

## Supporting information




**Supporting File 1**: advs77735‐sup‐0001‐SuppMat1.docx.

**Supporting File 2**: advs77735‐sup‐0002‐VideoS1.mp4.

**Supporting File 3**: advs77735‐sup‐0003‐VideoS2.mp4.

**Supporting File 4**: advs77735‐sup‐0004‐VideoS3.mp4.

**Supporting File 5**: advs77735‐sup‐0005‐VideoS4.mp4.
