# Supplementary material for: Decoding the Neural Circuit Underlying Context‐Cue Association in Retrieval of Morphine Reward Memory
Source: Adv Sci (Weinh). 2026 Sep 16:e77735. Online ahead of print. doi: 10.1002/advs.77735 (PMC13583090; doi:10.1002/advs.77735)
Supplement: Supplementary file 1 [file ADVS-9999-e77735-s004.docx]

**Decoding the Neural Circuit Underlying Context-Cue Association in** **Retrieval of Morphine Reward Memory**

**Zhifeng Shi, Guangying Li, Qian Yang, Rusun Liu, Linghan Gu, Mingmei Wang, John L. Waddington^*^, Fuhai Ji^*^, Xuechu Zhen^*^**

**Affiliations**

**Z. Shi, F. Ji, X. Zhen**

Department of Anesthesiology, First Affiliated Hospital of Soochow University, Suzhou 215123, Jiangsu, China

**Z. Shi, G. Li, Q. Yang, R. Liu, L. Gu, J. L. Waddington, X. Zhen**

Jiangsu Key Laboratory of Drug Discovery and Translational Research for Brain Diseases, College of Pharmaceutical Sciences, Soochow University, Suzhou 215123, Jiangsu, China

**Z. Shi, F. Ji, X. Zhen**

Institute of Anesthesiology, Soochow University, Suzhou 215123, Jiangsu, China

**J. L. Waddington**

School of Pharmacy and Biomolecular Sciences, RCSI University of Medicine and Health Sciences, Dublin, Ireland

**M. Wang, X. Zhen**

College of Biology & Food sciences, Suzhou University of Technology, Suzhou 215123, Jiangsu, China

^*^Correspondence: [jwaddington@rcsi.ie](mailto:jwaddington@rcsi.ie) (J.L.W.), jifuhai@suda.edu.cn (F.-H.J.), [zhenxuechu@suda.edu.cn](mailto:zhenxuechu@suda.edu.cn) (X.-C.Z.)

**Keywords:** glutamatergic projection, vCA1, dmPFC, microcircuit, reward memory retrieval

**Fig****S1. Manipulation of Glu^vCA1^**–**dmPFC circuit does not induce reward- or aversion-related behaviors.**

(A) Representative images showing the injection site in dmPFC (left), high-magnification images showing co-localization of mCherry+ neurons with CaMKIIα expression in vCA1 (middle) and quantification (right) of the percentage of mCherry+/CaMKIIα+ double-labeled neurons among mCherry+ neurons (n = 5; *t* (8) = 22.25, *P* < 0.001, unpaired Student’s *t* test). Scale bars 100 μm.

(B) Schematic illustration of the real-time place preference (RTPP) paradigm used for optogenetic manipulation of vCA1-dmPFC circuit.

(C) Representative tracking plots (left) showing mouse positions in the RTPP arena. Mice expressed mCherry, ChR2-EYFP or NpHR-mCherry in vCA1. Quantification of time (right) spent in the stimulation-paired compartment during RTPP testing revealed no significant differences across groups (mCherry, NpHR-mCherry groups: n = 11 each; ChR2-EYFP group: n = 9; *F* _(2, 56)_ = 0.26, *P* = 0.77, two-way ANOVA). Data are presented as mean ± SEM.

(D) Representative fluorescence images showing axonal terminals of ChR2-EYFP (left)-, NpHR-mCherry (middle)-, and mCherry (right)-expressing vCA1 neurons in dmPFC. Scale bars 100 μm.

**Fig S2. Inhibition of Glu^vCA1^**–**dmPFC circuit impairs morphine reward memory.**

(A) Experimental timeline and schematic diagram of the optogenetic stimulation protocol used in the conditioned place preference (CPP) paradigm.

(B) Left: Schematic for the optogenetic inactivation of vCA1 terminal projections in dmPFC. Right: Representative current-clamp recordings showing suppression of action potentials in NpHR-expressing vCA1 neurons upon 589 nm laser stimulation.

(C) Optogenetic inhibition of Glu^vCA1^-dmPFC pathway impaired morphine reward memory (mCherry group: n = 12; NpHR group: n = 10; *F* _(1, 20)_ = 7.01, *P* = 0.015, two-way ANOVA).

(D) Experimental timeline and schematic diagram of the chemogenetic manipulation protocol used in the CPP paradigm.

(E) Left: Schematic of chemogenetic inhibition of vCA1 terminal projections in dmPFC. Right: Representative current-clamp traces and quantification (bottom) showing decreased action potential firing in hM4Di-expressing vCA1 neurons following 100 pA current injection under CNO administration (*t* _(7)_ = 4.24, *P* < 0.001, paired Student’s *t* test).

(F) Chemogenetic inhibition of Glu^vCA1^-dmPFC pathway impaired morphine reward memory (N = 12 per group; *F* _(2, 44)_ = 5.80, *P* = 0.005, two-way ANOVA). Data are presented as mean ± SEM; **P* < 0.05, ***P* < 0.01, ****P* < 0.001.

**Fig S3. Inhibition of dmPFC-projecting Glu^vCA1^ neurons does not affect basal behaviors in mice.**

(A) Schematic showing optogenetic inhibition of vCA1 terminals in dmPFC (left) and experimental timeline of behavioral testing (right).

(B) Representative tracking plots from the novel object recognition (NOR) test (left) and quantification (right) showing that inhibition of dmPFC-projecting Glu^vCA1^ neurons did not alter preference for the novel object (n = 9 per group; *F* _(1, 16)_ = 27.79, *P* < 0.001, two-way ANOVA).

(C) Representative tracking plots from the open field test (OFT) (left) and quantification (right) showing that inhibition of dmPFC-projecting Glu^vCA1^ neurons did not affect locomotor activity (total distance: mCherry group: n = 12; NpHR group: n = 10; *F* _(1, 40)_ = 0.41, *P* = 0.53, two-way ANOVA). Data are presented as mean ± SEM; **P* < 0.05, ****P* < 0.001.

**Fig S4. Inhibition of vCA1 or dmPFC glutamatergic neurons does not affect NOR performance, while inhibition of dmPFC glutamatergic neurons alters locomotor activity in the OFT.**

(A, C) Representative traces of mouse locomotion during the NOR test following inhibition of glutamatergic neurons in vCA1 or dmPFC (left) and quantification showing no significant effect on novel object preference in either group (right) (vCA1: mCherry group, n = 8; NpHR group: n = 10; *F* _(1, 30)_ = 45.91, *P* < 0.001; dmPFC: mCherry group, n = 14; hM4Di group, n = 15; *F* _(1, 28)_ = 24.79, *P* < 0.001, two-way ANOVA).

(B, D) Left: Representative traces during the OFT. Right: Summary showing that inhibition of vCA1 glutamatergic neurons did not affect locomotor activity, whereas inhibition in dmPFC significantly reduced total distance. (vCA1: mCherry group, n = 8; NpHR group, n = 9; *F* _(1, 14)_ = 0.20, *P* = 0.66; dmPFC: mCherry group, n = 14; hM4Di group, n = 15; *F* _(1, 28)_ = 8.82, *P* = 0.001, two-way ANOVA). Data are presented as mean ± SEM; ****P* < 0.001.

**Fig S5. Activation of PV**+ **interneurons or inhibition of SST+ interneurons in** **dmPFC alters locomotor activity.**

(A, C) Representative traces of mouse locomotion during the novel object recognition (NOR) test following chemogenetic modulation of PV+ or SST+ interneurons activity (left) with quantification showing no significant effect on novel object preference in either group (right) (PV+ interneurons: DIO-EYFP, DIO-hM4Di groups, n = 13 each; DIO-hM3Dq group, n = 11; *F* _(1, 68)_ = 37.36, *P* < 0.001; SST+ interneurons: n = 10 per group; *F* _(1 , 54)_ = 26.78, *P* < 0.001, two-way ANOVA).

(B, D) Left: Representative traces from the open field (OF) test. Right: Quantification shows that chemogenetic activation of PV+ or inhibition of SST+ interneurons significantly reduced locomotor activity while inhibition of PV+ or activation of SST+ interneurons did not alter locomotor activity (PV+ interneurons: DIO-EYFP, DIO-hM4Di groups, n = 13 each; DIO-hM3Dq group, n = 11; *F* _(2, 68)_ = 4.95, *P* < 0.01; SST+ interneurons: DIO-EYFP, DIO-hM4Di groups, n = 10 each; DIO-hM3Dq group, n = 11 each; *F* _(2, 36)_ = 4.33, *P* = 0.021, two-way ANOVA). Data are presented as mean ± SEM; **P* < 0.05, ***P* < 0.01, ****P* < 0.001.

**Fig S6. Glu^vCA1^ inputs engage parallel excitatory and feedforward inhibitory pathways across different dmPFC neuronal subtypes.**

(A) Schematic of the Cre-dependent anterograde trans-monosynaptic tracing strategy.

(B) Representative images showing the injection site in vCA1 with AAV1-hSyn-Cre-EGFP. Scale bars 100 μm. (C) Representative images and quantification showing EGFP+ neurons within dmPFC co-localized with glutamatergic neurons and GABAergic interneurons (n = 5 per group; *t* _(8)_ = 14.30, *P* < 0.001, Student’s *t* test). Scale bars 100 μm.

(D) Schematic of simultaneous optogenetic activation of Glu^vCA1^ axon terminals and whole-cell patch-clamp recordings of EYFP-labeled neurons in dmPFC to examine synaptic connectivity across neuronal subtypes.

(E) Left: Representative current-clamp trace showing activation of EYFP-labeled Glu^dmPFC^ neuron by 473 nm blue light. Right: Representative traces of eEPSCs, recorded in the presence or absence of TTX and TTX + 4-AP recorded from Glu^dmPFC^.

(F) Similar recordings from a PV^dmPFC^ neuron show EPSCs in response to Glu^vCA1^ photostimulation.

(G) Same as (F) but for an SST^dmPFC^ neuron. Data are presented as mean ± SEM; ****P* < 0.001.

**Fig S7. Glu^vCA1^ inputs maintain E/I balance in dmPFC glutamatergic neurons but disrupt E/I balance in PV+ and SST+ interneurons through differential recruitment of local inhibitory circuitry.**

In pyramidal neurons activation of Glu^vCA1^ terminals significantly increased both the frequency and amplitude of EPSCs, along with an increase in IPSC frequency. However, the overall E/I ratio, in terms of both frequency and amplitude, remained unchanged, indicating a balanced enhancement of synaptic inputs. In contrast, PV+ interneurons exhibited a significant increase in both IPSC frequency and amplitude upon Glu^vCA1^ stimulation, leading to a marked reduction in both components of the E/I ratio. SST+ interneurons showed an increase in IPSC frequency without a significant change in amplitude, resulting in a selective decrease in the frequency component of their E/I ratio.
